# Coordination Effect-Promoted Durable Ni(OH)_2_ for Energy-Saving Hydrogen Evolution from Water/Methanol Co-Electrocatalysis

**DOI:** 10.1007/s40820-022-00940-3

**Published:** 2022-10-06

**Authors:** Guodong Fu, Xiaomin Kang, Yan Zhang, Xiaoqiang Yang, Lei Wang, Xian-Zhu Fu, Jiujun Zhang, Jing-Li Luo, Jianwen Liu

**Affiliations:** 1 Shenzhen Key Laboratory of Polymer Science and Technology, Guangdong Research Center for Interfacial Engineering of Functional Materials, College of Materials Science and Engineering, Shenzhen University, Shenzhen, 518060 People’s Republic of China; 2 School of Mechanical Engineering, University of South China, Hengyang, 421001 Hunan Province People’s Republic of China; 3 Institute for Sustainable Energy, College of Sciences, Shanghai University, Shanghai, 200444 People’s Republic of China

**Keywords:** Coordination effect, Methanol selective oxidation, NiMoO_4_, Formate, Energy-saving hydrogen production

## Abstract

Electrocatalytic water splitting is a viable technique for generating hydrogen but is precluded from the sluggish kinetics of oxygen evolution reactions (OER). Small molecule oxidation reactions with lower working potentials, such as methanol oxidation reactions, are good alternatives to OER with faster kinetics. However, the typically employed Ni-based electrocatalysts have poor activity and stability. Herein, a novel three-dimensional (3D)-networking Mo-doped Ni(OH)_2_ with ultralow Ni–Ni coordination is synthesized, which exhibits a high MOR activity of 100 mA cm^−2^ at 1.39 V, delivering 28 mV dec^−1^ for the Tafel slope. Meanwhile, hydrogen evolution with value-added formate co-generation is boosted with a current density of more than 500 mA cm^−2^ at a cell voltage of 2.00 V for 50 h, showing excellent stability in an industrial alkaline concentration (6 M KOH). Mechanistic studies based on density functional theory and X-ray absorption spectroscopy showed that the improved performance is mainly attributed to the ultralow Ni–Ni coordination, 3D-networking structures and Mo dopants, which improve the catalytic activity, increase the active site density and strengthen the Ni(OH)_2_ 3D-networking structures, respectively. This study paves a new way for designing electrocatalysts with enhanced activity and durability for industrial energy-saving hydrogen production.

**Supplementary Information:**

The online version contains supplementary material available at 10.1007/s40820-022-00940-3.

## Highlights

- A novel Ni(OH)_2_-based catalyst with ultralow Ni–Ni coordination is produced, exhibiting high activity (100 mA cm^−2^ at 1.39 V for methanol oxidation reactions) and outstanding stability in an industrial concentration electrolyte (over 500 mA cm^−2^ in 6 M KOH).
- Mechanistic studies show that the improved kinetics and durability are primarily due to ultralow Ni–Ni coordination, 3D-networking structures and the Mo dopant.

**Supplementary Information:**

The online version contains supplementary material available at 10.1007/s40820-022-00940-3.

## Graphical abstract

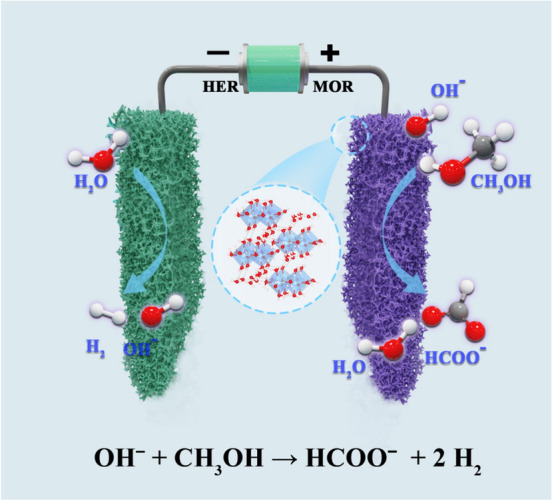

## Introduction

With the rising environmental problems and depletion of fossil fuels, the development and utilization of renewable energies have drawn extensive attention [1]. As clean and renewable energy, hydrogen is considered a potential replacement for fossil fuels owing to its high energy density and wide availability [2]. Electrocatalytic water splitting is a viable technique for obtaining hydrogen [3]. However, it is uneconomical to obtain hydrogen in a traditional electrocatalytic water splitting way. In the process, the cathode catalyzes hydrogen evolution reactions (HER) to produce hydrogen gas (H_2_), while the anode catalyzes oxygen evolution reactions (OER) to produce oxygen (O_2_) with high overpotential, leading to a low-economic-value anode product (O_2_). To solve these problems, the geometry and electronic structures of electrocatalysts have been widely studied to exploit low overpotential catalysts and improve economic efficiency. Such studies include heteroatom doping [4], defect modulation [5], tension modulation [6], surface self-reconstruction [7], metal-atom escape [8], heterointerface construction [9], tunable pore structures [10]. However, the overpotential of the best OER catalyst is still high at around 150 mV, whereas the overpotential of HER catalysts is close to 0 mV [11].

Alternatively, some novel anodic reactions have been used to replace OER, coupling HER reaction for energy-saving hydrogen production [12]. This kind of reaction is attractive because it does not involve OER, which is limited by the theoretical potential of 1.23 V. Most anodic reactions involve simple organic molecules, such as methanol [6, 13], ethanol [14, 15], glycerol [16, 17], urea [11, 18, 19], amine [20], furfural [21] and 5-hydroxymethylfurfural [22, 23] with low working potentials. Compared with traditional OER, anodic reactions based on simple organic molecule oxidation reduce the working voltage and exhibit better reaction kinetics. As the simplest alcohol, methanol can be easily produced by chemical or biomass industrial synthesis [24]. The synthesis of methanol is cheap compared to that of other organic matter (about 350 € per tonne). Furthermore, methanol exhibits very high solubility in water, and methanol oxidation reactions (MOR), which generate value-added formic acid and formate salts (about 539 € per tonne), have fast kinetics [25]. Thus, MOR is an ideal reaction to replace OER. Based on this, a novel electrolyzer can produce H_2_ and formate salts with low energy.

Recently, many MOR catalysts, including noble metals and earth-abundant transition metals, have been developed [26]. Noble metals, such as Pt [27], Ru [28], PtCu [29], PtNi [30], PtCo [31] and PtRu [32], have been reported for catalyzing MOR with low potential. However, CO poisoning and high cost limit their applications [33]. Earth-abundant transition metals catalyze MOR with low overpotential and produce formate with high Faradaic efficiency (> 90%) without CO_2_ emission [34]. Nowadays, Ni-based electrocatalysts have been widely studied for MOR. Ni(OH)_2_ [35, 36], NiCo–LDH [37], NiP_x_ [38], Ni_3_S_2_ [34, 39], NiMoO_4_ [40], Ni_2_P [41], Ni–MOF [42] and NiSe [43, 44] have excellent electrocatalytic activity. Furthermore, several methods, such as doping [45], phosphating [46], sulfurizing [47], anion intercalating [48] and anode–cathode exchange [49], have been employed to enhance the activity and durability of MOR electrocatalysts. Through these methods, the activity of electrocatalysts has been improved mainly through the following three pathways: (1) increase of the intrinsic activity of a single active site, (2) increase in the density of active sites; (3) enhancement of electronic conductivity.

For Ni-based materials, mechanistic studies have shown that Ni^2+^ is oxidized to Ni^3+^ species, which is a real active site for catalyzing methanol to formate [50]. Notably, surface evolution of Ni-based materials, such as oxidation, hydroxylation and reconstruction, usually occurs in the presence of an electrolyzer [34]. This phenomenon can achieve the aforementioned oxidation (Ni^2+^ → Ni^3+^), making them electrocatalytically active. In contrast, excessive evolution can deactivate the electrocatalysts [44]. The durability of Ni-based materials is still a challenge in alkaline hydrogenation evolution, especially at high current density in an industrial concentration (6 M KOH), which limits their applications. Thus, there is a need to develop durable Ni-based electrocatalysts at high current density in alkaline media with the industrial concentration.

In this study, we propose a facile strategy to enhance the kinetics and durability of Ni-based electrocatalysts by synthesizing Mo-doped 3D-networking Ni(OH)_2_ catalyst with ultralow Ni–Ni coordination from a NiMoO_4_·0.75 H_2_O precursor. The obtained electrocatalyst shows excellent MOR activity and high selectivity for value-added formate, especially at high current density in an industrial concentration. A current density of 100 mA cm^−2^ at 1.39 V is achieved for MOR, delivering 28 mV dec^−1^ for the Tafel slope. An assembled two-electrode electrolyzer generates 500 mA cm^−2^ at a cell voltage of 2.00 V with 90% Faradaic efficiency. Furthermore, electrolyzer operates for 50 h in an industrial concentration electrolyte (6 M KOH) without obvious deterioration. Mechanistic studies based on density functional theory (DFT) calculations and X-ray absorption spectroscopy (XAS) reveal that the improved kinetics and durability are mainly attributed to the (1) ultralow Ni–Ni coordination, which induces porosity in the structure, increasing the contact area and facilitating the reaction; (2) 3D-networking structures, which increase the density of the active sites; (3) uncompleted dissolution Mo, which strengthens the 3D-networking framework. This study paves a new way for designing electrocatalysts with enhanced activity and durability for industrial energy-saving hydrogen production.

## Experimental Section

### Synthesis of Electrocatalysts

**Scheme 1:**
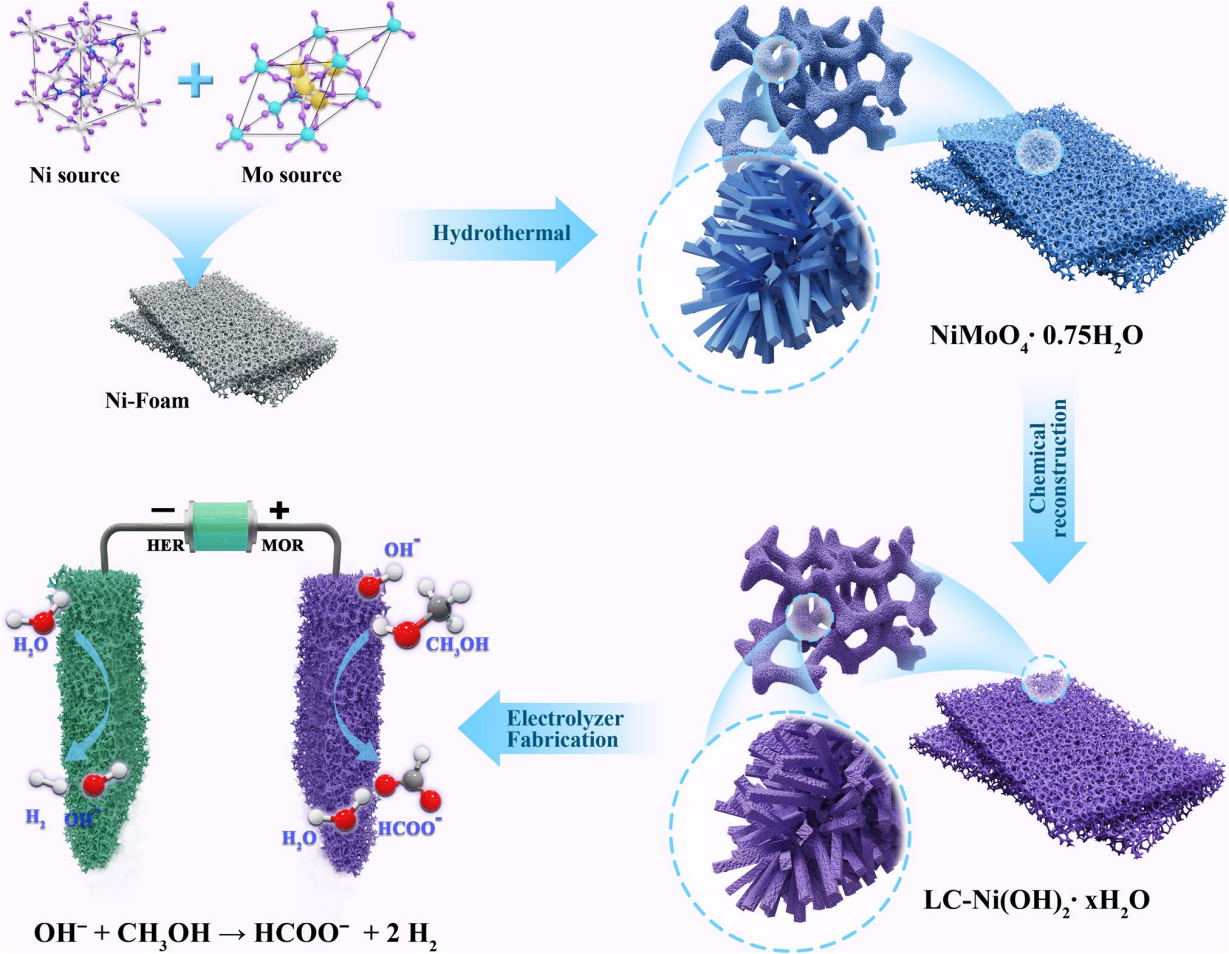
Preparation of low coordination Ni(OH)_2_·*x*H_2_O nanorod arrays and their assembly of the electrolytic cell coupling HER and MOR

Scheme 1 shows the procedure for preparing low coordination Ni(OH)_2_·*x*H_2_O nanorod arrays, i.e., LC–Ni(OH)_2_·*x*H_2_O. First, a hydrothermal approach was used to generate the precursor NiMoO_4_·0.75H_2_O on Ni foam using (NH_4_)_6_Mo_7_O_24_·4H_2_O and Ni(NO_3_)_2_·6H_2_O as Mo and Ni sources, respectively. Afterward, the grown NiMoO_4_·0.75 H_2_O nanorod arrays were put in a strong oxidizing and alkaline solution of NaOH and (NH_4_)_2_S_2_O_8_ for chemical reconstruction. With a simple chemical reconstruction, a novel Mo-doped 3D-networking Ni(OH)_2_ nanorod array, which was proved to be ultralow Ni–Ni coordinated, was successfully synthesized.

#### ***Preparation of Precursor NiMoO***_***4***_***·0.75 H***_***2***_***O***

A hydrothermal method was used to synthesize the precursor NiMoO_4_·0.75H_2_O. The commercial Ni foam (0.5 mm thickness) was first cleaned with 3 M HCl solution and ethanol for 30 min under ultrasonication, respectively. The Ni foam was dried under 60 °C after being cleaned with deionized (DI) water. Subsequently, in a Teflon autoclave heated to 150 °C for 6 h, 4 pieces of Ni foam (1 × 2 cm^2^) were immersed in 15 mL of H_2_O containing Ni(NO_3_)_2_·6H_2_O (0.04 M) and (NH_4_)_6_Mo_7_O_24_·4H_2_O (0.01 M). After washing with DI water, it was dried in 60 °C. Then, the NiMoO_4_·0.75H_2_O nanorod arrays were obtained.

#### Preparation of Anode and Cathode Electrocatalysts

The anode and cathode electrocatalysts are LC–Ni(OH)_2_·*x*H_2_O and Ni_4_Mo, respectively. To prepare of LC–Ni(OH)_2_·*x*H_2_O, 3.2 g NaOH and 1 g (NH_4_)_2_S_2_O_8_ were first dissolved in 16 and 8 mL DI water, respectively, to create solutions. Following that, both solutions were added in 18 mL DI water to generate a chemical reconstruction solution. Subsequently, the prepared precursor NiMoO_4_·0.75 H_2_O nanorod arrays were immersed in the solution for 30 min before being washed with DI water and dried at 40 °C to obtain LC–Ni(OH)_2_·*x*H_2_O. In addition, samples with reconstruction times of 10 and 20 min were also prepared and named as LC–Ni(OH)_2_·*x*H_2_O-10 and LC–Ni(OH)_2_·*x*H_2_O-20, respectively. For the preparation of Ni_4_Mo, the NiMoO_4_·*x*H_2_O nanorod arrays were heated at 500 ℃ for 2 h in a H_2_ (5%)/Ar (95%) atmosphere and then obtained the Ni_4_Mo electrocatalyst.

#### ***Preparation of Ni(OH)***_***2***_

Typically, a hydrothermal process is used to create Ni(OH)_2_ on Ni foam. In this instance, 60 mL of DI water was used to dissolve 1.732 g of NiCl_2_·6H_2_O and 1.1 g of urea. To create a homogeneous solution, the aqueous solution was thoroughly stirred. One piece of the treated Ni foam was then placed into the prepared solution, transferred to a stainless steel autoclave lined with Teflon and kept at 120 °C for 12 h. The finished product was thoroughly cleaned and repeatedly sonicated with DI water and ethanol after naturally cooling to room temperature. The sample was then dried overnight at 60 °C in the air.

### Characterization

The morphologies of electrodes were characterized by field-emission scanning electron microscopy (FESEM, SU-70) and field-emission transmission electron microscopy (FETEM, JEM-F200). In addition, X-ray diffraction (XRD), X-ray photoelectron spectroscopy (XPS) and the Inductively Coupled Plasma-Optical Emission Spectrometer (ICP-OES) were used for detailed analysis. The XRD patterns were recorded using the Bruker D8 Advance (Cu Kα, 50 kV and 360 mA). The XPS was conducted using a Thermo Scientific™ K-Alpha™^+^ spectrometer equipped with a monochromatic Al Kα X-ray source (1486.6 eV) operating at 100 W. Samples were analyzed under vacuum (*P* < 10^−8^ mbar) with a pass energy of 150 eV (survey scans) or 25 eV (high-resolution scans). All peaks were calibrated with C 1* s* peak binding energy at 284.8 eV for adventitious carbon. The ICP-OES was recorded by Agilent 5110. Furthermore, Raman spectra and XAS were used to deeply study the fine structure of the synthesized electrocatalysts. For Raman spectra, it was recorded by Horiba: HR Evolution with a 633-nm laser, whereas the XAS was collected by employing synchrotron radiation light source at BL12B2 beam line of the National Synchrotron Radiation Research Center (NSRRC) in SPring 8 (Japan) at room temperature. The detailed analysis of the XAS is listed in supporting information.

### Electrochemical Measurements

All electrochemical measurements were taken with a electrochemical workstation (CHI760E, CH instruments Inc., Shanghai) at room temperature. The electrochemical measurements were taken in a three-electrode system. The catalyst-loaded Ni foam (1 × 1 cm^2^) was used as working electrode, and the Hg/HgO was used as reference electrode. Pt sheet (1 × 1 cm^2^) was used as a counter electrode, while a graphite rod was used in case of HER to avoid potential contamination of Pt. 1 M KOH with or without 0.5 M methanol was used as electrolytes, and polarization curves were collected at a scan rate of 5 mV s^−1^. The potential drop (*i*R) loss due to the solution/system resistance was applied according to the equation: *E*_corr_ = *E*_mea_–*i*R. All potentials presented in this work were calibrated to the reversible hydrogen electrode (RHE) according to the equation: *E*_RHE_ = *E*_Hg/HgO_ + 0.059pH + 0.098. Double-layer capacitance (*C*_dl_) of the as-prepared electrode was measured by cyclic voltammetry in a potential range of 0.978 − 1.078 V vs RHE at scan rate of 10 − 50 mV s^−1^. A large current density MOR experiment was carried out in a two-electrode system. With 6 M KOH and 3 M methanol serving as the electrolyte, the LC − Ni(OH)_2_·*x*H_2_O loaded on Ni foam (1 × 0.5 cm^2^) was employed as the anode, while the Ni_4_Mo served as the cathode.

### Product Analysis

Ion chromatography (Technology Co. Ltd., Qingdao, China), which was equipped with organic anion columns containing the leachate of 2.4 mmol Na_2_CO_3_ and 6 mmol NaHCO_3_, was employed for the quantification of products from electrochemical oxidation of methanol. Before chronoamperometry measurements, 100 uL of electrolyte was collected and diluted with DI water with a ratio of 1:100. The measurement of each sample was repeated three times, and the concentration of formate ion was calibrated based on standard solutions with known concentrations. The detailed calculations for the Faradaic efficiency (FE) and energy consumptions are listed in the supporting information.

### Computational Details

Using the Vienna ab-initio Simulation Package (VASP), the spin-polarized DFT calculations were carried out for NiMoO_4_·0.75H_2_O, Ni(OH)_2_ and LC − Ni(OH)_2_·*x*H_2_O [51]. For these calculations, the electronic exchange and correlation effects were described using the Perdew–Burke–Ernzerhof (PBE) functionals [52]. The lattice parameters for NiMoO_4_·0.75H_2_O were obtained from previous report [53], i.e., a = 6.82063 Å, b = 6.90884 Å, c = 9.34338 Å, α = 76.18636°, β = 83.84972°, γ = 74.03878°. And the experimental lattice parameters for Ni(OH)_2_ were used for the calculations, i.e., a = b = 3.114 Å, c = 4.617 Å, α = β = 90°,  γ = 120°. A 6 × 6 × 6 k-point mesh was used for NiMoO_4_·0.75H_2_O, and a 10 × 10 × 10 k-point mesh was used for Ni(OH)_2_. The cutoff energy was set as 600 eV, and the criterion for the geometry optimization was set as 0.01 eV Å^−1^ for the force. To account for the strong electron correlation, the on-site Hubbard correlations (*U*) were considered with *U*_eff_ = 5.5 eV for Ni [54] and dispersion correction was used following the previous studies [55]. For the lattice optimization of Ni(OH)_2_·2.75H_2_O and Ni(OH)_2_·2.50H_2_O, the cell shape was fixed, and only the size was optimized.

## Results and Discussion

### Materials Characterization

**Fig. 1:**
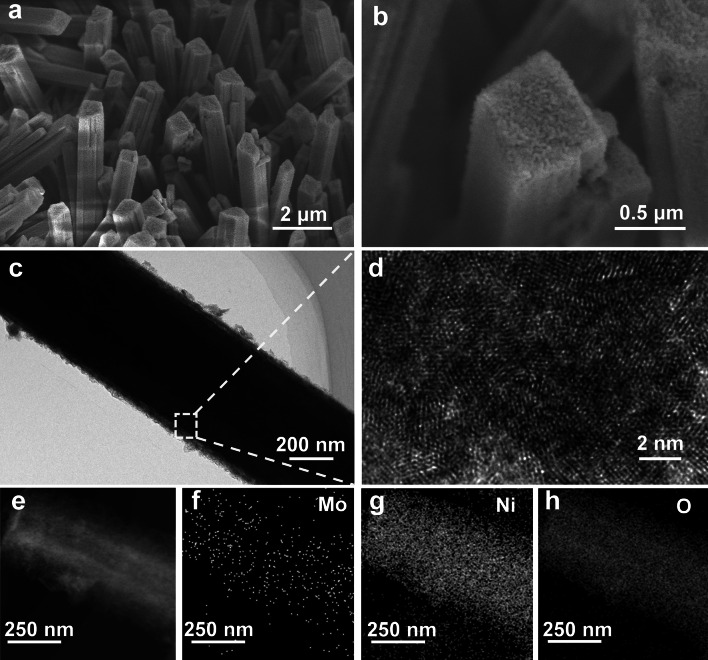
**a**, **b** SEM of LC–Ni(OH)_2_ *x*H_2_O. **c** TEM of LC–Ni(OH)_2_ *x*H_2_O**, d** HRTEM of LC–Ni(OH)_2_ *x*H_2_O. **e** High-angle annular dark-field (HADDF) of LC–Ni(OH)_2_ *x*H_2_O. **f–h** Elemental mapping images of LC–Ni(OH)_2_·*x*H_2_O

The SEM, high-resolution TEM (HRTEM) and energy-dispersive X-ray spectroscopy (EDS) are performed to investigate the changes in the morphology and composition of the NiMoO_4_·0.75H_2_O precursor and LC–Ni(OH)_2_·*x*H_2_O nanorod array. Notably, the transition products from the precursors to the final electrocatalysts, namely LC–Ni(OH)_2_·*x*H_2_O-10 and LC–Ni(OH)_2_·*x*H_2_O-20, which are chemically constructed in 10 and 20 min, are also analyzed in detail to determine the chemical evolution process. The SEM images of the NiMoO_4_·0.75 H_2_O precursor (Fig. S1a-b) show well-crystallized cuboids with sizes ranging from 0.5 to 1.5 μm and lengths of ~ 10 μm. As chemical reconstitution proceeds, the structure of the micron cuboids could be observed, but the previously smooth surface changes to a crude surface for LC–Ni(OH)_2_·*x*H_2_O-10 (Fig. S2), LC–Ni(OH)_2_·*x*H_2_O-20 (Fig. S3) and LC–Ni(OH)_2_·*x*H_2_O (Fig. 1a-b). HRTEM reveals that a stubbly substance grows on the surface of the electrocatalyst (Fig. 1c). With higher magnification, the lattice fringes become irregular, and dark spots could be observed (Fig. 1d), indicating that the electrocatalyst becomes more amorphous due to chemical remodeling processes. The EDS element mapping images (Fig. 1e-h) show a decrease in the Mo element content, which is lower than that of NiMoO_4_·0.75H_2_O (Fig. S1), LC–Ni(OH)_2_·*x*H_2_O-10 (Fig. S2) and LC–Ni(OH)_2_·*x*H_2_O-20 (Fig. S3). It indicates that most of the Mo atoms are dissolved. However, some small amount Mo may be still in the LC–Ni(OH)_2_·*x*H_2_O, acting as dopants. As a result, the precursor NiMoO_4_·0.75H_2_O has evolved into LC–Ni(OH)_2_·*x*H_2_O with small amount of Mo as dopants.

**Fig. 2:**
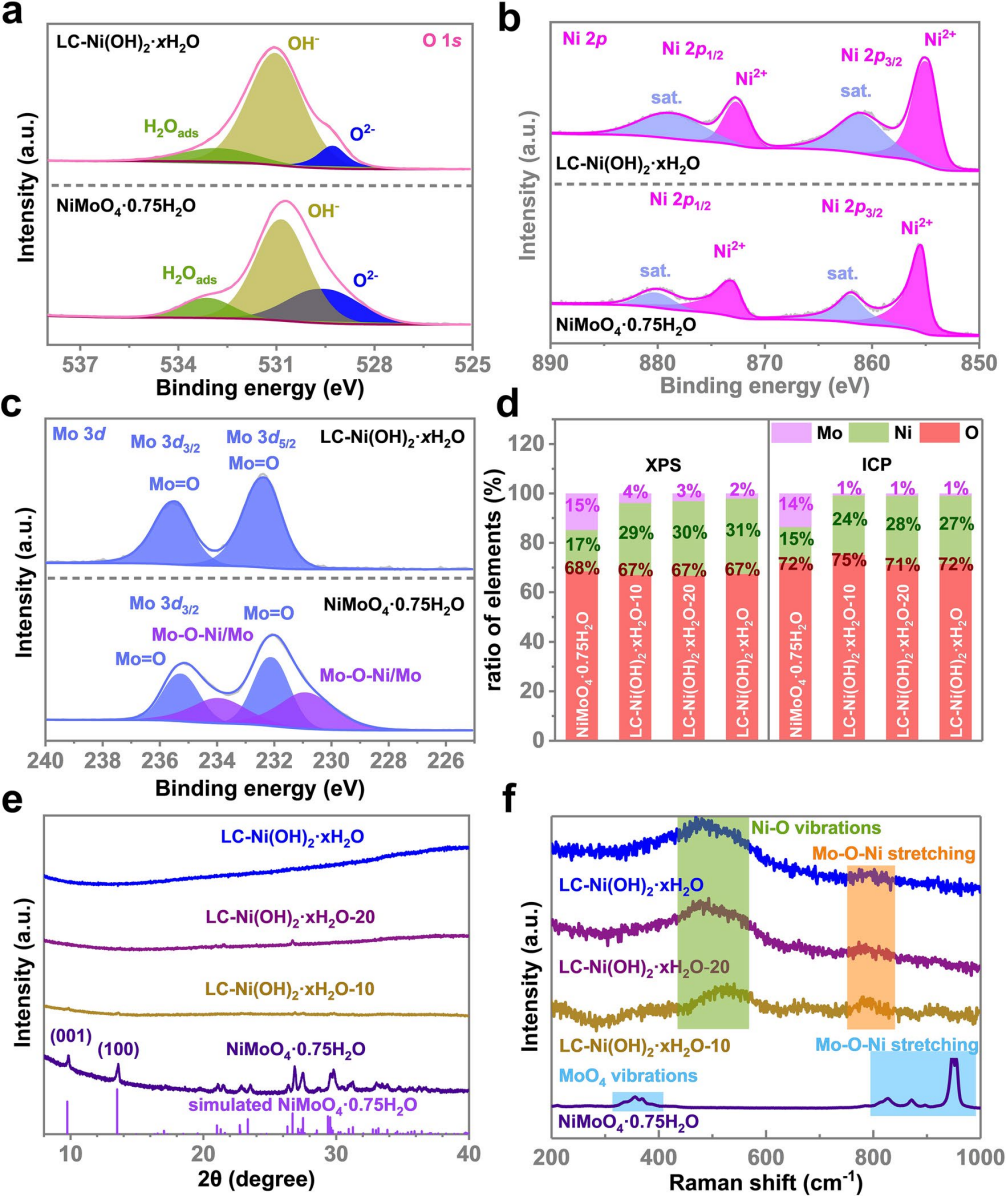
**a-c** High-resolution O 1*s*, Ni 2*p* and Mo 3*d* XPS spectra of NiMoO_4_·0.75 H_2_O and LC–Ni(OH)_2_ ·*x*H_2_O. **d** Ratio of elements by XPS and ICP-OES, **e** XRD of NiMoO_4_·0.75H_2_O, LC–Ni(OH)_2_·*x*H_2_O–10, LC–Ni(OH)_2_·*x*H_2_O-20 and LC–Ni(OH)_2_·*x*H_2_O. **f** Raman spectra of NiMoO_4_·0.75H_2_O, LC–Ni(OH)_2_·*x*H_2_O–10, LC–Ni(OH)_2_·*x*H_2_O-20 and LC–Ni(OH)_2_·*x*H_2_O

The chemical reconstruction is also studied by XPS and ICP-OES. As shown in Fig. 2a, the XPS O 1*s* spectrum of NiMoO_4_·0.75 H_2_O and LC–Ni(OH)_2_·*x*H_2_O could be deconvoluted into three surface components at 533.1, 530.0 and 529.5 eV, which are ascribed to the adsorbed water molecules (H_2_O), surface hydroxide (OH^−^) and lattice O^2−^ species, respectively [56]. According to the components analysis (Table S1), the percentage of the OH peak in O 1*s* increases, while the percentage of the O^2−^ peak decreases, indicating that the linking Mo atoms dissolve as MoO_4_^2−^, resulting in the formation of LC–Ni(OH)_2_·*x*H_2_O [57]. The comparison for XPS of precursor NiMoO_4_·0.75 H_2_O, final product LC–Ni(OH)_2_·*x*H_2_O and transition products LC–Ni(OH)_2_·*x*H_2_O-10, LC–Ni(OH)_2_·*x*H_2_O-20 (Fig. S4) confirms the observation. With the evolution of the reconstruction, the signals of Mo dramatically decline, while the signals of Ni and O hold steady, indicating the dissolution of Mo atoms. Notably, the XPS shows all these samples do not contain any substantial impurities except the carbon, which is added for calibration of the energy. Figure 2b-c compares the high-resolution XPS spectra of Ni 2*p* and Mo 3*d*. Ni 2*p*_3/2_ and 2*p*_1/2_ show prominent peaks at 855.5 and 873.2 eV, respectively, for NiMoO_4_·0.75 H_2_O, which are attributed to Ni^2+^ in the oxide. However, for LC–Ni(OH)_2_·*x*H_2_O, the Ni^2+^ peak shifts from 855.5 to 855.4 eV. As Ni^2+^ and Ni^3+^ (with peaks at 858.0 and 876.2 eV) usually occur at the same time [58], we try to locate the peaks for Ni^3+^. However, these peaks cannot be observed in this case. The main reason could be the special structures of NiMoO_4_·0.75 H_2_O, LC–Ni(OH)_2_·*x*H_2_O, both of which are primarily Ni^2+^. The peaks for Mo 3*d*_5/2_ and 3*d*_3/2_ at 232.1, 235.1 and 230.9, 233.9 eV could be deconvoluted into Mo=O and Mo–O–M (M=Ni or Mo) components for NiMoO_4_·0.75 H_2_O. However, that of Mo=O shifts from 232.1 to 232.4 eV for LC–Ni(OH)_2_·*x*H_2_O. The energy shift may be attributed to the change in the chemical environment, i.e., the Mo dissolution to form MoO_4_^2−^, during the chemical reconstruction. In addition, the disappearance of the Mo–O–M peak at 230.9 and 233.9 eV for LC–Ni(OH)_2_·*x*H_2_O further confirms the collapse of NiMoO_4_·0.75H_2_O crystal structure. XPS and ICP confirm that NiMoO_4_·0.75 H_2_O is composed of Ni, Mo and O, with a molar ratio of approximately 1:1:5, respectively (Fig. 2d). In comparison, ICP–OES and XPS reveal that the Ni to O ratio is 1:3, and the Mo content of LC–Ni(OH)_2_·*x*H_2_O is 1%, indicating the dissolution of Mo atoms in NiMoO_4_·0.75 H_2_O.

XRD is conducted to analyze the crystal structures of NiMoO_4_·0.75H_2_O and LC–Ni(OH)_2_∙*x*H_2_O (Fig. 2e). Some characteristic peaks of NiMoO_4_·0.75H_2_O are observed, but the signal gradually decreases until it disappears when the NiMoO_4_·0.75H_2_O transition to LC–Ni(OH)_2_·*x*H_2_O. Despite the lack of a standard XRD card for NiMoO_4_·0.75H_2_O, all of the peaks in the 5–40° range can be matched with the simulated diffraction pattern of nickel molybdate hexahydrate (NiMoO_4_·0.75 H_2_O), which has been systematically studied [53]. Based on these results, we confirm that the precursor is indeed NiMoO_4_·0.75 H_2_O. Some very weak signals are observed in the 20 − 30° range for the transition products, which may be attributed to unconverted NiMoO_4_·0.75 H_2_O. However, no obvious signals for LC–Ni(OH)_2_·*x*H_2_O are observed, indicating the complete conversion from NiMoO_4_·0.75H_2_O to LC–Ni(OH)_2_·*x*H_2_O. Moreover, the disappearance of signals indicates that LC–Ni(OH)_2_∙*x*H_2_O is amorphous, as shown in the HRTEM image (Fig. 1d).

Furthermore, Raman spectroscopy is conducted to analyze the precursor, transition products and final electrocatalyst. The Raman spectra (Fig. 2f) show the peaks of MoO_4_ vibrations (355 cm^−1^) and Ni–O–Mo stretching (800–1000 cm^−1^) for precursor NiMoO_4_·0.75H_2_O [59]. However, the MoO_4_ vibrations (355 cm^−1^) and Ni–O–Mo stretching (800–1000 cm^−1^) almost disappear with the evolution from NiMoO_4_·0.75H_2_O to LC–Ni(OH)_2_·*x*H_2_O. Meanwhile, the peaks of Ni–O vibrations (474 and 554 cm^−1^) [60] appear and become more prominent. These two peaks are characteristic of Ni(OH)_2_ species [61], indicating that NiMoO_4_·0.75H_2_O was converted into Ni(OH)_2_-based species. Additional Mo–O–Ni stretching (~ 788 cm^−1^) ascribed to LC–Ni(OH)_2_·*x*H_2_O and the transition products are observed, which could be attributed to undissolved Mo in the structure. The chemical environment is dramatically altered because the compound containing NiMoO_4_ as the framework (NiMoO_4_·0.75H_2_O) is transformed into the compound having Ni(OH)_2_ as the framework (LC–Ni(OH)_2_·*x*H_2_O), resulting in a shift in the frequency of Mo–O–Ni stretching from 800–1000 to 788 cm^−1^. Furthermore, the spectrum reveals that as the chemical reconstruction progresses, more Mo dissolves, causing the Mo–O–Ni stretching peaks to weaken and even vanish.

### Electrochemical Performance

**Fig. 3:**
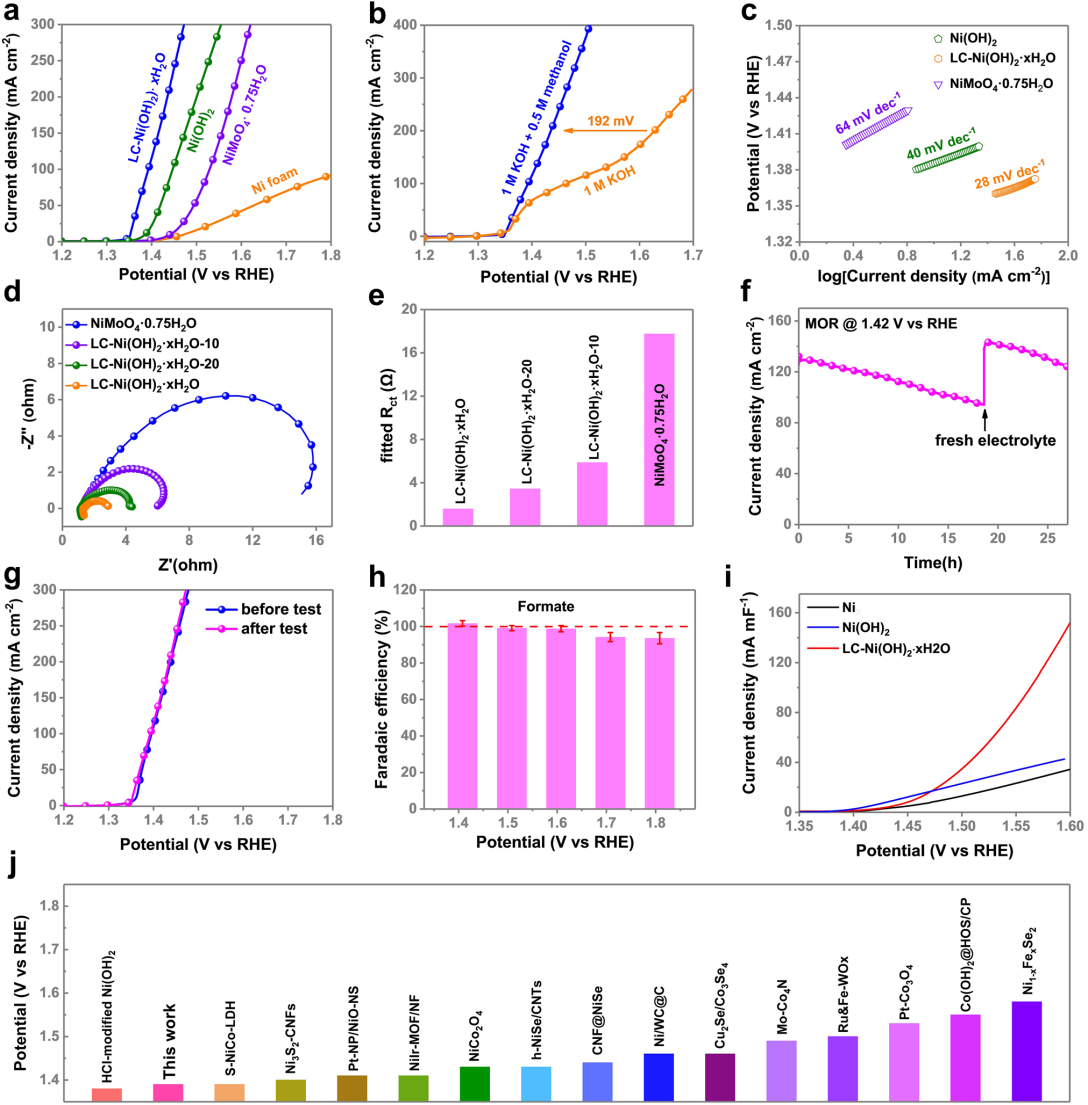
**a** Linear sweep voltammetry (LSV) curves of LC–Ni(OH)_2_ ·*x*H_2_O, Ni(OH)_2_, NiMoO_4_ ·0.75H_2_O and Ni foam in 1 M KOH and 0.5 M methanol. **b** LSV curve of LC–Ni(OH)_2_ ·*x*H_2_O with and without 0.5 M methanol. **c** Tafel slope of LC–Ni(OH)_2_ ·*x*H_2_O, Ni(OH)_2_, NiMoO_4_ ·0.75H_2_O and Ni foam. **d-e** EIS spectra and fitted *R*_ct_ of LC–Ni(OH)_2_ ·*x*H_2_O, Ni(OH)_2_, NiMoO_4_ ·0.75H_2_O and Ni foam. **f** *i–t* curve of the stability test before and after the stability test for LC–Ni(OH)_2_ ·*x*H_2_O. **g** LSV curve before and after the stability test for LC–Ni(OH)_2_ ·*x*H_2_O. **h** Changed 500C Faradaic efficiency of MOR catalyzed by LC–Ni(OH)_2_ ·*x*H_2_O at different potentials. **i** *C*_dl_-normalized LSV curve of catalysts in 1 M KOH and 0.5 M methanol. **j** Comparison of the performance of the electrocatalyst synthesized herein and that of previously reported organic molecule oxidation catalysts. The detailed data are listed in Table S2 of the Supporting Information

In the presence of 0.5 M methanol, the polarization curves of LC–Ni(OH)_2_·*x*H_2_O, Ni(OH)_2_, NiMoO_4_·0.75H_2_O and Ni foam are compared in 1 M KOH. The onset potential of Ni(OH)_2_·*x*H_2_O against reversible RHE for methanol oxidation processes is 1.35 V, which is better than that of NiMoO_4_·0.75H_2_O and Ni(OH)_2_ (Fig. 3a). Furthermore, at a potential of 1.43 V (vs. RHE), LC–Ni(OH)_2_·*x*H_2_O shows a current density of 200 mA cm^−2^, which is 192 mV lower than that of traditional OER (Fig. 3b), demonstrating the high potential of LC–Ni(OH)_2_·*x*H_2_O for energy-saving hydrogen production. Notably, an oxidation peak appeared at 1.39 V vs. RHE in OER (Fig. 3b), which can be assigned to the oxidation of Ni^2+^ to Ni^3+^ [62]. To verify this, a cycle voltammetric measurement of LC–Ni(OH)_2_·*x*H_2_O is taken (Fig. S5), which confirms the observation. Figure 3c reveals that the Tafel slope for LC–Ni(OH)_2_·*x*H_2_O is 28 mV dec^−1^, which is substantially slower than that of NiMoO_4_·0.75 H_2_O (64 mV dec^−1^) and Ni(OH)_2_ (40 mV dec^−1^), showing that the dissolution of Mo species significantly accelerates the kinetics of MOR.

To further examine the electrode kinetics and the ionic and charge transport resistance of MOR, electrochemical impedance spectroscopy (EIS) is used at 1.37 V (vs. RHE) in 1 M KOH and 0.5 M methanol, with the associated Nyquist plots (Fig. 3d). The equivalent circuit is composed of a resistor representing the Ohmic resistance (*R*_s_) and a parallel combination, including a resistor reflecting the charge transfer resistance (*R*_ct_) and a constant-phase element (CPE-1) [63]. Compared to LC–Ni(OH)_2_·*x*H_2_O-20 (3.4 Ω), LC–Ni(OH)_2_·*x*H_2_O-10 (5.8 Ω) and NiMoO_4_·0.75H_2_O (17.7 Ω), LC–Ni(OH)_2_·*x*H_2_O exhibits the least R_ct_ of 1.6 Ω, indicating enhanced electron transport (Fig. 3e). In the presence of 0.5 M methanol, the stability of LC–Ni(OH)_2_∙*x*H_2_O is examined in 1 M KOH at 1.42 V (vs. RHE) for 100,000 s. The current density declines from ~ 130 to 95 mA cm^−2^ due to methanol consumption, as shown in Fig. 3f. After refreshing the electrolyte, the current density increases back to ~ 130 mA cm^−2^ without obvious deterioration, showing its high potential for energy-saving hydrogen production. There is no significant difference in the LSV curves of LC–Ni(OH)_2_·*x*H_2_O before and after the stability test (Fig. 3g). In addition, the SEM image (Fig. S6a-b), Raman shift (Fig. S6c) and ICP–OES (Fig. S6d) after the stability test also show no significant change, which confirms that it exhibits excellent stability. Ion chromatography (Fig. S7) is used to study the Faradaic efficiency of MOR catalyzed by LC–Ni(OH)_2_·*x*H_2_O at various potentials. The Faradaic efficiency declines from 100% (1.4 V versus RHE) to 95% (1.8 V vs. RHE) as the potential increased (Fig. 3h). This can be attributed to the significant likelihood of OER competition. Further, the double-layer capacitance (*C*_dl_) measured by cyclic voltammetry (Fig. S8) is employed to compare the electrochemical active surface area (ECSA). However, ECSA = *C*_dl_/*C*_s_, where *C*_s_ is a constant and a common factor. So, it is reasonable to use *C*_dl_ to replace ECSA in the qualitative comparison of intrinsic activity. Ni(OH)_2_ has a much higher *C*_dl_ (2.99 mF cm^−2^) than LC–Ni(OH)_2_∙*x*H_2_O (1.85 mF cm^−2^) (Fig. S9). However, the *C*_dl_ normalized activity of LC–Ni(OH)_2_·*x*H_2_O is higher than that of Ni(OH)_2_, indicating that it has a much higher density of active sites (Fig. 3i). We compare its performance to that of recently reported catalysts for methanol/water co-electrolysis with higher current density at 100 mA cm^−2^, and we find that LC–Ni(OH)_2_·*x*H_2_O is similar to the best HCl-modified Ni(OH)_2_ and more active than others, including NiSe, NiFe, NiMoO_4_ and Ni_3_S_2_ (Fig. 3j).

### Electronic Structure and Mechanisms Analysis

HRTEM and XRD reveal that the synthesized catalyst has an amorphous structure, and XPS reveals that the amorphous structure has the similar structure as Ni(OH)_2_, but its morphologies and XRD peaks do not match those of Ni(OH)_2_. However, it outperforms Ni(OH)_2_; thus, there is a need to understand the structure of the catalyst to accelerate the high-performance Ni-based electrocatalysts design.

**Fig. 4:**
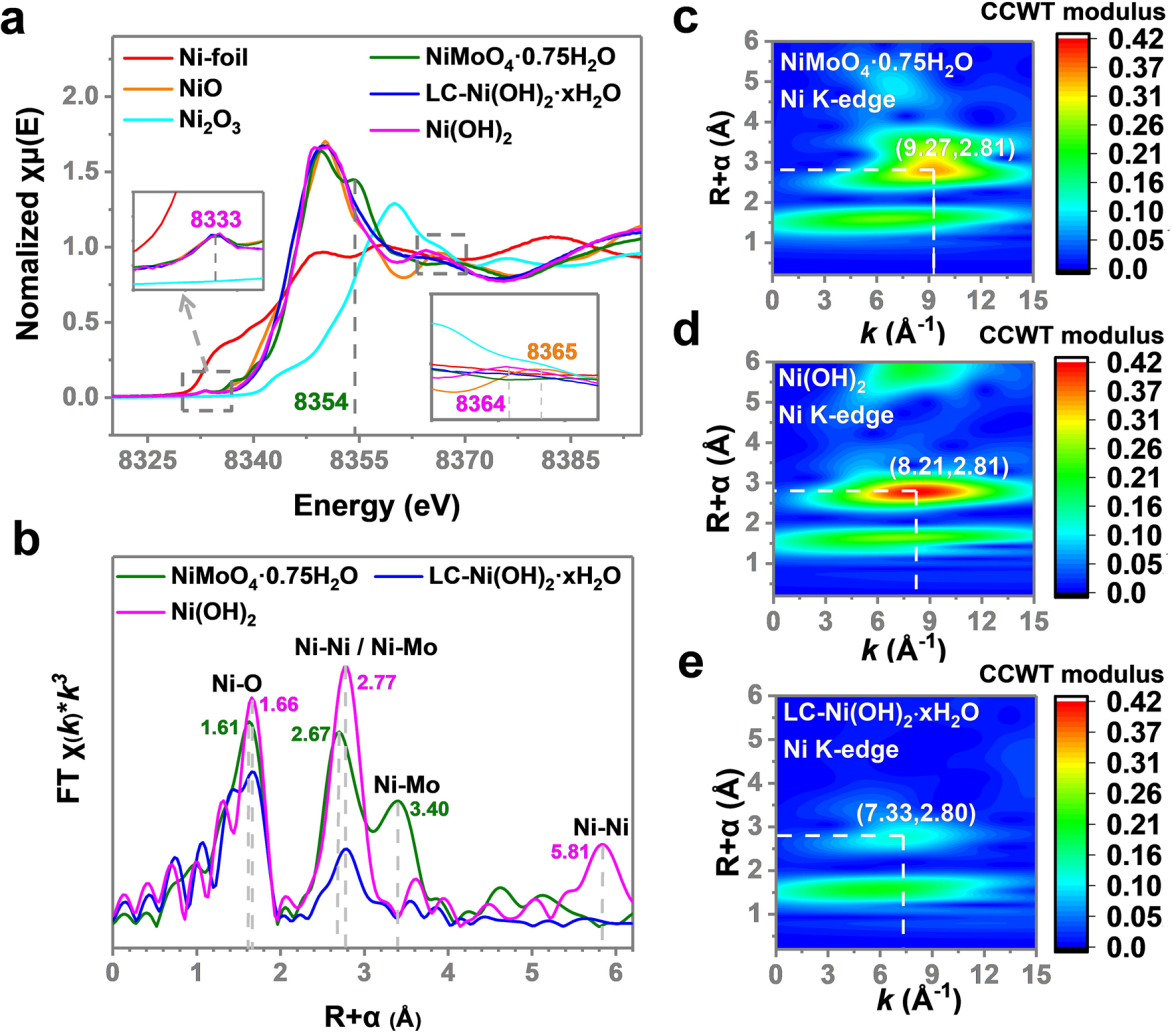
**a** Ni K-edge images of NiMoO_4_ ·0.75H_2_O, LC–Ni(OH)_2_ ·*x*H_2_O, Ni(OH)_2_, Ni foil, NiO and Ni_2_O_3_. **b** Fourier transform EXAFS in R space and **c–e** wt-EXAFS for NiMoO_4_·0.75H_2_O, Ni(OH)_2_ and LC–Ni(OH)_2_ ·*x*H_2_O

Figure 4a shows the normalized X-ray absorption near-edge spectroscopy (XANES) of NiMoO_4_·0.75H_2_O and LC–Ni(OH)_2_·*x*H_2_O with Ni foil, NiO, Ni_2_O_3_ and Ni(OH)_2_ for comparison. The curves for the Ni foil (Ni^0^) and Ni_2_O_3_ (Ni^3+^) differ from those of the other Ni^2+^, including NiMoO_4_·0.75H_2_O, LC–Ni(OH)_2_·*x*H_2_O, NiO and Ni(OH)_2_. The Ni^2+^ species showed a pre-edge peak at 8333 eV, which is attributed to the 1* s* → 3*d* quadrupole transition [64]. In addition, NiMoO_4_·0.75H_2_O and NiO showed characteristic peaks at 8354 and 8365 eV, respectively. Thus, the curves for LC–Ni(OH)_2_·*x*H_2_O are very similar to that of Ni(OH)_2_, indicating that they have similar properties, which is in well agreement with the XPS observations.

Figure 4b shows the Fourier transformed curves of the extended X-ray absorption fine structure (EXAFS) in R space. There is a peak at 1.66 Å for LC–Ni(OH)_2_·*x*H_2_O and Ni(OH)_2_, which could be assigned to Ni–O paths. For NiMoO_4_·0.75H_2_O, however, there is a shifted peak at 1.61 Å for Ni–O paths probably due to the coupling between Mo–O and Ni–O bonds. In addition, LC–Ni(OH)_2_·*x*H_2_O and Ni(OH)_2_ show a peak at 2.77 Å, which could be assigned to the first shell of the Ni–Ni path. For NiMoO_4_·0.75H_2_O, because the Ni–Ni and Ni–Mo paths are indistinguishable, a little smaller shift is observed at 2.67 Å, which is attributed to the Ni–Ni/Ni–Mo paths. Also, NiMoO_4_·0.75H_2_O showed a peak at 3.41 Å, which could be assigned to the long Ni–Mo path in NiMoO_4_·0.75H_2_O. Notably, Ni(OH)_2_ shows a characteristic peak at 5.8 Å, which could be assigned to the second shell of the Ni–Ni path, whereas LC–Ni(OH)_2_·*x*H_2_O shows no peak at 5.8 Å. This further indicates the unique Ni–Ni coordination structure for this novel electrocatalyst.

Wavelet transform EXAFS (wt-EXAFS) of the electrocatalysts is shown in Fig. 4c–e, which show four, three and two shells, respectively. The maximum for these samples is at ~ 2.81 Å on the R-axis and at 9.27, 8.21 and 7.33 Å^−1^ on the *k*-axis. The peak of LC–Ni(OH)_2_·*x*H_2_O at 2.81 Å shifts from the high- to the low-k region compared with that of the other two species, indicating that the decrease in the peak (Fig. 4b) is attributed to the absence of paths for heavy atoms, such as Mo and Ni, rather than light atoms, such as O. This further indicates the dissolution of Mo species and the low coordination number of the Ni–Ni path.

**Fig. 5:**
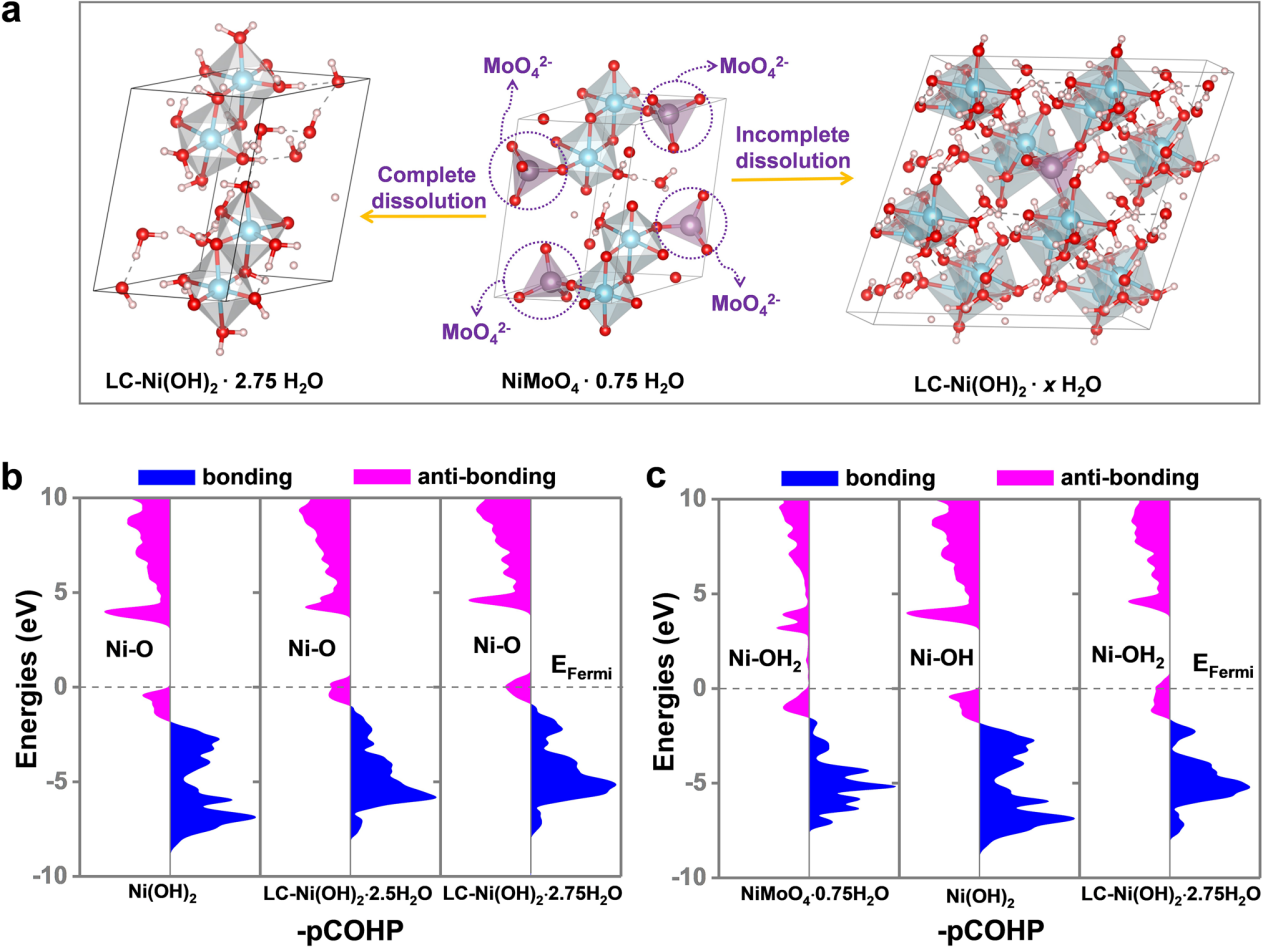
**a** Illustration for the formation of LC-Ni(OH)_2_·2.75H_2_O and Mo-doped LC–Ni(OH)_2_·*x*H_2_O from NiMoO_4_·0.75H_2_O. **b** Crystal orbital Hamilton population (COHP) for all Ni–O bonds of Ni(OH)_2_, LC–Ni(OH)_2_·2.50H_2_O and LC–Ni(OH)_2_·2.75H_2_O. **c** Crystal orbital Hamilton population (COHP) for active Ni–O bonds of NiMoO_4_∙0.75H_2_O, Ni(OH)_2_ and LC–Ni(OH)_2_∙2.75H_2_O. -pCOHP refers the negative projected COHP

Based on physical and electronic characterizations, we propose the chemical construction mechanisms (Fig. 5a). With the strong oxidation of alkaline media, the OH^−^ group attacks the Mo − O bonds, resulting in the dissolution of Mo atoms as MoO_4_^2−^. If all Mo atoms dissolve, and the structural framework does not alter much, a novel Ni(OH)_2_-based compound with low coordination for Ni − Ni, i.e., LC − Ni(OH)_2_·2.75H_2_O, can be obtained. A single unit of this unique Ni(OH)_2_-based compound contains an average of 2.75 crystalline H_2_O molecules. There are two kinds of crystalline H_2_O present here: H_2_O that directly coordinates with Ni atoms and that which does not coordinate with Ni atoms but instead forms hydrogen bonds with other H_2_O molecules. Non-coordinated H_2_O, on the other hand, can diffuse, resulting in the low coordination structure LC − Ni(OH)_2_·2.50H_2_O. These two Ni(OH)_2_-based molecules have nearly identical structures. However, DFT calculations revealed that they have different optimum lattice parameters: the optimized lattice parameters for LC − Ni(OH)_2_·2.50H_2_O are approximately 2% smaller than those for LC − Ni(OH)_2_·2.75H_2_O (Fig. S10). Thus, the crystalline structure contains uncertain crystalline H_2_O molecules, making it amorphous. As a result, XRD could not obtain the crystalline structure. Furthermore, because Mo cannot be completely dissolved, residual Mo remains as a dopant in the form of Mo–O–Ni (Fig. 5a). Thus, the newly formed catalyst can be deduced as Mo-doped Ni(OH)_2_ containing uncertain crystalline water.

To verify this deduction, we perform simulations using these models and the EXAFS data. Surprisingly, the experimental EXAFS results are well reproduced (Fig. S11 and Table S3). The signals for the Ni–Mo bonds disappear during the processes, which is in agreement with the ICP–OES, XPS, Raman and EXAFS results, indicating that Mo species in NiMoO_4_·0.75H_2_O are selectively dissolved, forming a novel nickel hydroxide species. Compared with the well-crystallized Ni(OH)_2_ with a Ni–Ni coordination number of 6, LC–Ni(OH)_2_·*x*H_2_O showed an ultralow Ni–Ni coordination number of 1.5 (Table S3 and Fig. S12). In addition, according to the ICP and XPS results, approximately 1 to 2% Mo remained in the crystalline structure. Because Mo is linked to Ni in the NiMoO_4_·0.75H_2_O structure, we infer that it links with Ni in the novel Ni(OH)_2_ structures through bridged oxygen. Based on these results, we conclude that a new Mo-doped Ni(OH)_2_ containing uncertain crystalline water, namely, LC–Ni(OH)_2_·*x*H_2_O, is formed.

Crystal orbital Hamilton populations (COHPs) are analyzed based on DFT calculations to further understand the performance enhancement. As shown in Fig. 5b, COHP describes the bonding and antibonding of Ni–O bonds in Ni(OH)_2_, LC–Ni(OH)_2_·2.5H_2_O and LC–Ni(OH)_2_·2.75H_2_O. As the number of H_2_O molecules increases, the composition of antibonding orbitals below the Fermi level decreases, lowering the system's energy. This means that the addition of a water molecule strengthens the entire Ni–O bonds of the system, making the catalyst more stable. For comparison, we calculated COHP for the active Ni sites, i.e., the Ni–O bonds (Ni–OH_2_) formed by Ni and H_2_O molecules for NiMoO_4_ and LC–Ni(OH)_2_·2.75H_2_O (Fig. 5c) since they are loosely bonded and more easily attacked by OH^−^ groups than other Ni–O bonds (Table S4). Surprisingly, the composition of the Ni–O antibonding orbitals in either NiMoO_4_·0.75H_2_O or LC–Ni(OH)_2_·2.75H_2_O is similar to that of Ni(OH)_2_ below the Fermi level. This indicates that although the overall stability is improved, the activity of the active site is still comparable to that of Ni(OH)_2_. However, LC–Ni(OH)_2_·2.75H_2_O has a 3D-networking structure and hydrogen bonding H_2_O, which can expose more active sites by releasing the H_2_O and enhance the density of the active sites; thus, the total activity is higher than that of Ni(OH)_2_, which explains why LC–Ni(OH)_2_·*x*H_2_O has a low ECSA (Fig. S6b) but high normalized ECSA activity, as observed in the experiment (Fig. 3i).

**Fig. 6:**
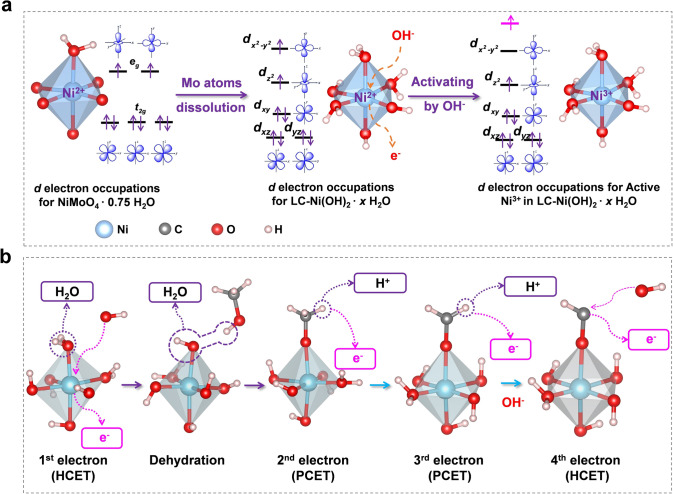
**a** Illustration of *d* electron occupation for NiMoO_4_ ·0.75H_2_O, LC–Ni(OH)_2_·2.75H_2_O and activated LC–Ni(OH)_2_ ·2.75H_2_O. **b** Illustration of methanol oxidation mechanisms with 4-electron processes

Further, we investigated the *d* orbital occupations of the active Ni sites to understand the activity enhancement for LC–Ni(OH)_2_·*x*H_2_O (Fig. 6a). Five of the six Ni–O bonds at the active Ni sites in NiMoO_4_·0.75H_2_O originate from Ni–O–Mo bonds, and the other originates from Ni–H_2_O bonding. Thus, the *d* orbitals have relatively high symmetry, with one group consisting of double degenerated *e*_*g*_ orbitals and the other consisting of three degenerated *t*_*2g*_ orbitals. When LC–Ni(OH)_2_·*x*H_2_O is formed after Mo is dissolved out, three Ni–O bonds in the active Ni center originate from OH groups, and the other three originate from H_2_O molecules, which significantly changes the electron state of its *d* orbitals, thereby breaking the symmetry and lowering the energy of the system. When OH^−^ attacks the Ni active center and releases an electron, i.e., through a hydroxide-ion-coupling electron transfer (HCET) process, one of the high occupied *d* electrons is stimulated to a higher energy antibonding orbital, increasing the antibonding composition of the Ni–O bond. Therefore, the valence Ni atom shifts from + 2 to + 3, activating the Ni for electrocatalytic reactions.

The mechanism of methanol oxidation is depicted in Fig. 6b, where the four-electron process of OER is replaced by a four-electron process of methanol oxidation. The four-electron methanol oxidation process involves two HCET and two proton-coupled electron transfer (PCET) processes. As aforementioned, when OH^−^ attacks the active center of Ni atoms, Ni^2+^ is oxidized to Ni^3+^ through the HCET process. Following that, methanol reacts with the hydroxide attached to Ni^3+^ to form –OCH_3_. In the presence of an electric field, –OCH_3_ releases a proton and an electron through the PCET process to form –OCH_2_. Similarly, in the presence of an electric field, –OCH_2_ releases a proton and an electron to form –OCH. Then, OH^−^ continues to attack –OCH through an HCET process, releasing an electron and forming formic acid. The formic acid quickly neutralizes with the alkali in a strongly alkaline environment (6 M KOH) and then generates formate.

Collectively, Mo species in NiMoO_4_·0.75 H_2_O nanorods are selectively dissolved, forming Mo-doped Ni(OH)_2_. Due to the low coordination effect, amorphous and 3D-networking LC–Ni(OH)_2_·*x*H_2_O is formed. For such a special structure, there are more H_2_O and OH^−^ groups around Ni instead of Ni atoms, which increase the contact area between OH^−^ and CH_3_OH and the catalytic active center; thus, the electrochemical properties are increased. Meanwhile, it maintains the Ni(OH)_2_ activity for a single site; therefore, overall, the LC–Ni(OH)_2_·*x*H_2_O activity is higher than that of Ni(OH)_2_. In addition, part of the Mo that remains and fails to dissolve plays a role in doping therein. It exists in the form of a Mo–O–Ni chemical bond, which is stronger than the hydrogen bond, making the active Ni(OH)_2_ unit combine more firmly and improve the stability. Therefore, the coordination effect improves not only the activity but also the stability.

### Electrolytic Cell Performance

**Fig. 7:**
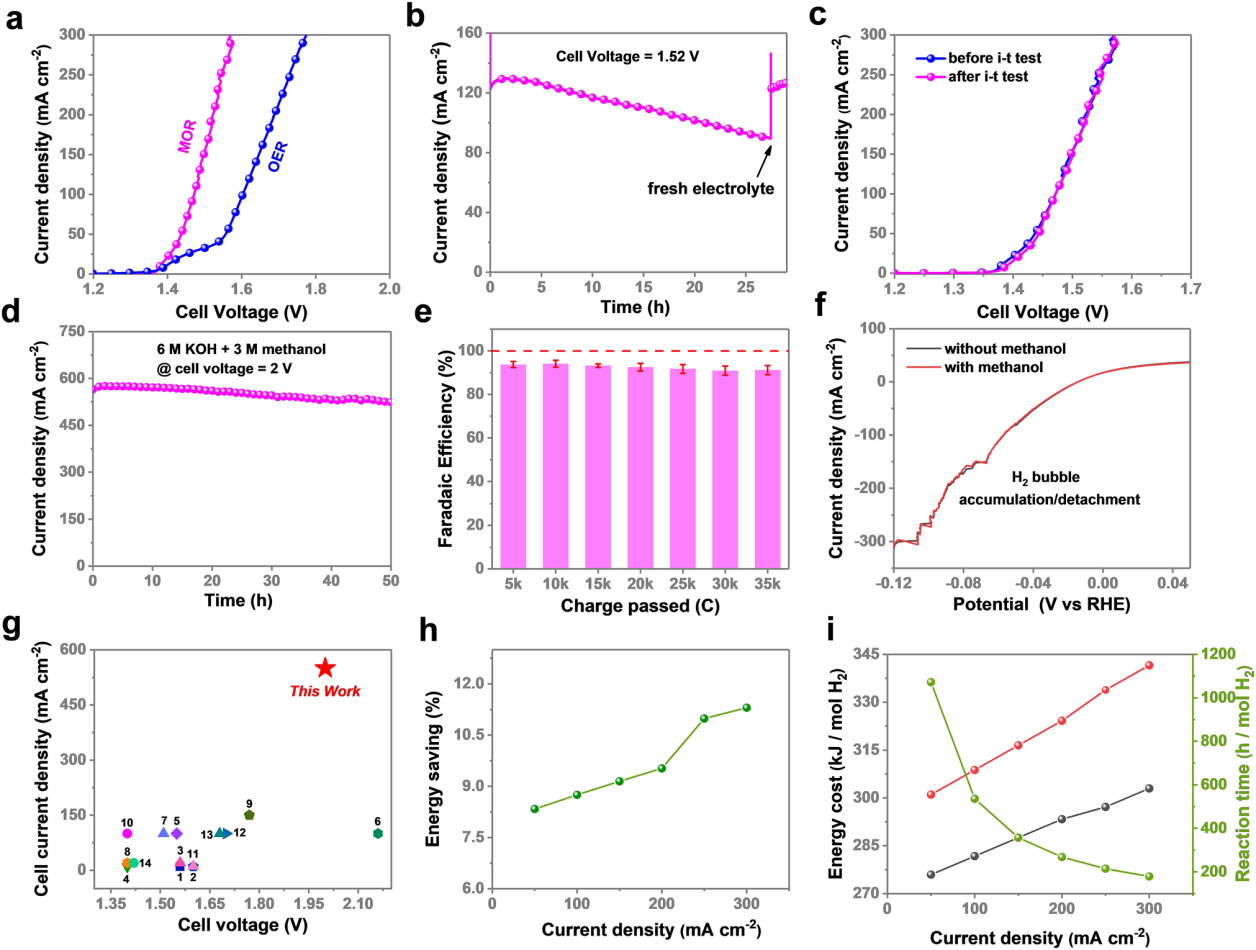
**a** LSV curves for the two-electrode consisting of Ni_4_Mo–MoO_2_ and LC–Ni(OH)_2_·*x*H_2_O in 1 M KOH with and without 0.5 M methanol. **b** *i–t* curves in 1 M KOH and 0.5 M methanol, **c** LSV curves before and after stability tests and **d** *i–t* curves in 6 M KOH and 3 M methanol for the Ni_4_Mo–MoO_2_ || LC–Ni(OH)_2_·*x*H_2_O cell. **e** Faradaic efficiency during the stability test in 6 M KOH and 3 M methanol. **f** LSV curve of Ni_4_Mo–MoO_2_ in 1 M KOH with and without 0.5 M methanol. **g** Performance comparison with recently reported oxidation of organic molecules coupled with HER. The detailed data are listed in Table S5 of the Supporting Information. **h** Percentage energy saving for hydrogen production at different current densities when OER is replaced by methanol oxidation reaction. **i** Energy cost for generating the same amount of H_2_ (1 mol) integrated with OER or MOR at constant current densities. The right tick label indicates the required reaction time. The energy costs and percentage energy savings were calculated based on the data in Fig. 7a

A two-electrode system with Ni_4_Mo-MoO_2_ as the cathode and LC–Ni(OH)_2_·*x*H_2_O as the anode is used to produce hydrogen and value-added formate simultaneously. The Ni_4_Mo–MoO_2_ is synthesized by heating NiMoO_4_·0.75H_2_O in an H_2_/Ar atmosphere following the literature [65], and the SEM image shows that Ni_4_Mo alloy nanoparticles grow on the surface of the MoO_2_ nanorods (Fig. S13a-b). The XRD confirms the structure of Ni_4_Mo-MoO_2_ nanoparticles as indicated in Fig. S13c. After forming the electrolytic cell, the polarization curves of 0.5 M methanol and no methanol show a significant difference in 1 M KOH (Fig. 7a). In the presence of methanol, MOR dominates, whereas OER dominates in the absence of methanol. The stability of the two-electrode cell system is examined for 100,000 s (Fig. 7b-c). In the beginning, a voltage of 1.52 V was applied to the cell, resulting in a current density of 130 mA cm^−2^. Because of the consumption of methanol and OH^−^, the current density gradually decreased to 95 mA cm^−2^. After refreshing the electrolyte, the current density increased back to 130 mA cm^−2^. The coincidence of the LSV curves before and after the stability test also indicates that the two-electrode system is stable (Fig. 7c). To investigate the application of this system under the industrial conditions of high current density and high alkaline electrolyte, we perform electrolysis in 6 M KOH and 3 M methanol at 2.00 V (cell voltage) with over 500 mA cm^−2^ (without *i*R correction) for more than 50 h. The current density remains almost constant throughout the tests (Fig. 7d), indicating high activity and stability in the industrial concentration. Furthermore, the Faradaic efficiency remains above 90% (Fig. 7e). When we compare the HER activity of Ni_4_Mo–MoO_2_ with and without methanol (Fig. 7f), we discover that the enhanced kinetics of methanol/water co-electrolysis is mostly due to LC–Ni(OH)_2_·*x*H_2_O.

Further, we compared the performance of the obtained electrocatalyst with that of recently reported catalysts for small organic molecule co-electrolysis (Fig. 7g). The Ni_4_Mo–MoO_2_ || LC–Ni(OH)_2_·*x*H_2_O cell obtained herein is the only catalysts could produce a current density of 500 mA cm^−2^ for small organic molecule co-electrolysis to the best of our knowledge, indicating that the obtained catalyst has potential industrial applications. In the case of the two-electrode system of Ni_4_Mo–MoO_2_ || LC–Ni(OH)_2_·*x*H_2_O in 1 M KOH and 0.5 M methanol, only 1.49 V is required to obtain a current density of 150 mA cm^−2^ (Fig. 7a). In the absence of methanol, a higher cell voltage of 1.64 V is required to obtain the same current density. This voltage gap between the cells with and without methanol indicates that energy can be saved in hydrogen production by replacing OER with MOR. For 1 mol H_2_, the energy cost for overall water splitting (HER||OER) is 316 kJ, whereas that of the methanol value-added boost H_2_ production (HER||MOR) is only 287 kJ, indicating that 8.3–11.2% energy can be saved at current densities of 50–300 mA cm^−2^, as demonstrated in Fig. 7h-i. Furthermore, MOR produces formate (about 550 € per tonne), which is more expensive than methanol (about 350 € per tonne), making this reaction more valuable.

In summary, electrolytic cells composed of LC–Ni(OH)_2_·*x*H_2_O and Ni_4_Mo–MoO_2_ can co-electrolyze methanol/water to produce hydrogen at industrial concentrations with low-energy demand owing to the enhanced activity and durability of the LC–Ni(OH)_2_·*x*H_2_O electrocatalyst. This proposed hydrogen production method can save 8.3–11.2% energy compared to the traditional direct water electrolysis. In addition, the proposed reaction would be more useful because it can produce high-value chemical formate. Thus, this strategy is economically viable for practical industrial applications.

## Conclusions

A novel Mo-doped Ni(OH)_2_ containing uncertain H_2_O molecules electrocatalyst LC–Ni(OH)_2_·*x*H_2_O was synthesized through the selective dissolution of Mo species from NiMoO_4_·0.75H_2_O. It shows enhanced kinetics and durability for energy-saving hydrogen production with the co-generation of a highly selective value-added product (formate) for water/methanol co-catalysis. TEM, XPS and EXAFS revealed that dense NiMoO_4_·0.75H_2_O is *in situ* converted to 3D-networking LC–Ni(OH)_2_·*x*H_2_O. Meanwhile, the hydrogen evolution with value-added formate co-generation is boosted at a large current density of more than 500 mA cm^−2^ and a cell voltage of 2.00 V. The Faradaic efficiency is more than 90% at the current density of more than 500 mA cm^−2^ with excellent stability for 50 h in a high-concentration electrolyte (6 M KOH). Although XRD could not reveal the structure of LC–Ni(OH)_2_·*x*H_2_O because the composition of crystalline H_2_O is uncertain, the fine structure is resolved using XAS and DFT based on the environmental variables, such as the Ni bond length and coordination number. Further mechanistic studies based on DFT revealed that the improved kinetics and durability are mainly attributed to the ultralow Ni–Ni coordination effect for active Ni sites, which results in 3D-networking structures. The ultralow Ni–Ni coordination, 3D-networking structures and Mo dopants improve the intrinsic catalytic activity, increase the active site density and strengthen the binding of 3D-networking structure, respectively. By replacing OER with MOR at the anode, the voltage of the cell consisting of Ni_4_Mo–MoO_2_ as the cathode and LC–Ni(OH)_2_·*x*H_2_O as the anode decreased from 1.64 to 1.49 V, significantly lowering the energy consumption for hydrogen production. This study paves a new way for realizing low-energy electrolysis of water in industrial alkaline conditions for hydrogen production.

## Supplementary Information

Below is the link to the electronic supplementary material.

Supplementary file1 (PDF 1978 kb)
