# Supplementary material for: Coordination Effect-Promoted Durable Ni(OH)2 for Energy-Saving Hydrogen Evolution from Water/Methanol Co-Electrocatalysis
Source: Nanomicro Lett. 2022 Oct 6;14:200. doi: 10.1007/s40820-022-00940-3 (PMC9537394; doi:10.1007/s40820-022-00940-3)
Supplement: Supplementary file 1 — Supplementary file1 (PDF 1978 kb) [file 40820_2022_940_MOESM1_ESM.pdf]

Supporting Information for

Coordination-Effect-Promoted Durable $Ni(OH)_2$ for Energy-Saving Hydrogen Evolution from Water/Methanol Co-Electrocatalysis

Guodong Fu [1], Xiaomin Kang [1, 2], Yan Zhang [1], Xiaoqiang Yang [1], Lei Wang [1], Xian-Zhu Fu [1], Jiujun Zhang [3], Jing-Li Luo [1] and Jianwen Liu [1, *]

[1]Shenzhen Key Laboratory of Polymer Science and Technology, Guangdong Research Center for Interfacial Engineering of Functional Materials, College of Materials Science and Engineering, Shenzhen University, Shenzhen, 518060, P. R. China

[2]School of Mechanical Engineering, University of South China, Hengyang, 421001, Hunan Province, P. R. China

[3]Institute for Sustainable Energy, College of Sciences, Shanghai University, Shanghai, 200444, P. R. China

*Corresponding author. E-mail: jwliu@szu.edu.cn (Jianwen Liu)

S1 General Characterization

The XAS data were analyzed using Demeter version 0.9.26 [S1]. The Energy calibration was performed with a Ni-foil standard by shifting all spectra to a glitch in the incident intensity. Extended X-ray absorption fine structure (EXAFS) spectra were fitted using the FEFF 6.0 code.

S2 Electrochemical Measurements

The potential drop (*i*R) loss due to the solution/system resistance were applied according to the equation:

$$E_{corr} = E_{mea} - iR \tag{S1}$$

All potentials presented in this work were calibrated to the reversible hydrogen electrode (RHE) according to the equation:

$$E_{RHE} = E_{Hg/HgO} + 0.059pH + 0.098 \tag{S2}$$

The HER and MOR equations are as follows:

$$2H_2O + 2e^- \rightarrow H_2 + 2OH^-$$

and

$$CH_3OH + 5OH^- \rightarrow HCOO^- + 4H_2O + 4e^-$$

Overall:

$$CH_3OH + OH^- \rightarrow HCOO^- + 2H_2$$

S3 Product Analysis

The faradaic efficiency (FE) of formate is calculated as follows:

$$FE_{formate} = \frac{4nF}{Q} \times 100\% \tag{S3}$$

Where *n* is the mol of the formate; *F* is the faraday constant, 96485 C mol^{-1}; and Q is the total charge passed (C).

The energy consumption saving percentage of hydrogen at different current densities is calculated as follows:

$$P_{energy\ saving} = \frac{U_{OER}-U_{MOR}}{U_{OER}} \times 100\% \qquad (S4)$$

Where U_{OER} and U_{MOR} is the cell voltage of HER||OER cell and HER||MOR cell at certain current density, respectively.

The energy cost for generating the same amount of H_2 integrated with OER or MOR is calculated as follow:

$$E = UnF \qquad (S5)$$

Where U is the cell voltage; n is the mol of H_2; F is the faraday constant, 96485 C mol^{-1};

The reaction time for generating H_2 integrated with OER or MOR are calculated as follow:

$$t = \frac{nF}{I} \qquad (S6)$$

Where I is the cell current density; n is the mol of H_2.

S4 Supplementary Tables and Figures

Table S1 Percentage contents for $NiMoO_4 \cdot 0.75H_2O$ and LC–$Ni(OH)_2 \cdot xH_2O$ calculated from the XPS peaks

Chemical state	Samples	Peak area	Peak ratio
H_2O	$NiMoO_4 \cdot 0.75H_2O$	38564.05	3.50
H_2O	LC–$Ni(OH)_2 \cdot xH_2O$	32259.91	12.68
OH^-	$NiMoO_4 \cdot 0.75H_2O$	187840.24	65.75
OH^-	LC–$Ni(OH)_2 \cdot xH_2O$	197780.9	77.74
O^{2-}	$NiMoO_4 \cdot 0.75H_2O$	59298.47	20.76
O^{2-}	LC–$Ni(OH)_2 \cdot xH_2O$	24358.78	9.58

Table S2 Comparison of the Tafel slopes and Potential at 100 mA cm^{-2} of LC–$Ni(OH)_2 \cdot xH_2O$ and the reported catalysts applied for the electrocatalytic oxidation in 1M KOH

Catalysts	Tafel scope (mV dec^{-1})	Potential @ 100 mA cm^{-2} (V vs RHE)	Raw materials	Refs.
Pt–Co_3O_4	138	1.53	methanol	J. Mater. Chem. A **2021**, 9, 6316
CNF@NiSe	24	1.44	methanol	J. Mater. Chem. A **2019**, 7, 25878
Ni/WC@C	–	1.46	methanol	Chem. Mater. **2022**, *34*, 959
$NiCo_2O_4$	–	1.43	methanol	J. Energy Chem. **2019**, *29*, 136
$Ni_{1-x}Fe_xSe_2$	–	1.58	methanol	Small **2021**, *17*, 2006623
HCl–modified $Ni(OH)_2$	17.6	1.36	methanol	Appl. Catal. B **2021**, *281*, 119510
S–NiCo–LDH	38.7	1.39	methanol	J. Mater. Chem. A **2022**, *10*, 1329
Ni_3S_2–CNFs	27.3	1.40	methanol	Nano Energy **2021**, *80*, 105530
Pt–NP/NiO–NS	27	1.41	methanol	Chem. Eng. J. **2021**, *411*, 128292
Ru&Fe–WOx	32	1.50	methanol	Appl. Catal. B **2021**, *296*, 120359

Mo–Co_4N	42	1.49	methanol	J. Mater. Chem. A **2021**, *9*, 21094
Cu_2Se/Co_3Se_4	87	1.46	methanol	Appl. Catal. B **2021**, *285*,119800
h–NiSe/CNTs	39.2	1.43	methanol	Adv. Funct. Mater. **2021**, *31*, 2008812
$Co(OH)_2$@HOS/CP	71	1.55	methanol	Adv. Funct. Mater. **2020**, *30*, 1909610
NiIr–MOF/NF	17.7	1.41	methanol	Appl. Catal. B **2022**, 300, 120753
LC–$Ni(OH)_2{\cdot}xH_2O$	28	1.39	methanol	This Work

Table S3 Parameter for EXAFS fitting

Sample	Path	N	S_0^2	σ^2 (10^{-3} Å^2)	e_0 (eV)	R(Å)	R-Factor (%)	K-space range (Å^{-1})	R-space range (Å)
$NiMoO_4 \cdot 0.75 H_2O$ Ni Kα	Ni–O	6	0.9(1)	6(1)	–1(1)	2.03(1)	2.4	3–15	1–4
	Ni–Ni	2		3(1)		2.96(1)			
	Ni–Mo	3		6(1)		3.48(1)			
	Ni–Mo	3		6(2)		3.82(2)			
$Ni(OH)_2$ Ni Kα	Ni–O	6	0.9(1)	6(1)	–4(1)	2.06(1)	1.8	3–15	1–3.4
	Ni–Ni	6		6(1)	–4(1)	3.11(1)			
LC–$Ni(OH)_2 \cdot 2.75H_2O$ Ni Kα	Ni–O	6	0.9(1)	8(1)	–3(1)	2.04(1)	3.2	3–15	1–3.4
	Ni–Ni	1.5		8(1)		3.07(1)			
LC–$Ni(OH)_2 \cdot 2.5H_2O$ Ni Kα	Ni–O	6	0.9(1)	8(1)	–3(1)	2.04(1)	3.9	3–15	1–3.4
	Ni–Ni	1.5		6(1)		3.07(1)			

Table S4 Selective distances (Å) for optimized LC–$Ni(OH)_2{\cdot}2.75H_2O$ and LC–$Ni(OH)_2{\cdot}2.5H_2O$

**$Ni(OH)_2{\cdot}2.75H_2O$**		**$Ni(OH)_2{\cdot}2.50H_2O$**	
Ni_1–O_1: 2.10	Ni_3–O_{11}: 2.14	Ni_1–O_1: 2.10	Ni_3–O_{11}: 2.14
Ni_1–O_2: 2.19	Ni_3–O_{12}: 2.16	Ni_1–O_2: 2.15	Ni_3–O_{12}: 2.10
Ni_1–O_3: 2.06	Ni_3–O_{13}: 2.05	Ni_1–O_3: 2.06	Ni_3–O_{13}: 2.05
Ni_1–O_4: 2.06	Ni_3–O_{14}: 2.15	Ni_1–O_4: 2.04	Ni_3–O_{14}: 2.12
Ni_1–O_5: 2.05	Ni_3–O_{15}: 2.00	Ni_1–O_5: 2.05	Ni_3–O_{15}: 2.10
Ni_1–O_6: 2.14	Ni_3–O_{16}: 2.05	Ni_1–O_6: 2.21	Ni_3–O_{16}: 2.05
Ni_2–O_3: 2.05	Ni_4–O_{13}: 2.12	Ni_2–O_3: 2.01	Ni_4–O_{13}: 2.06
Ni_2–O_7: 1.99	Ni_4–O_{17}: 2.06	Ni_2–O_7: 2.05	Ni_4–O_{17}: 2.05
Ni_2–O_8: 2.32	Ni_4–O_{18}: 2.06	Ni_2–O_8: 2.25	Ni_4–O_{18}: 2.17
Ni_2–O_9: 2.07	Ni_4–O_{19}: 2.11	Ni_2–O_9: 2.05	Ni_4–O_{19}: 2.13
Ni_2–O_6: 2.13	Ni_4–O_{16}: 2.03	Ni_2–O_6: 2.16	Ni_4–O_{16}: 2.09
Ni_2–O_{10}: 2.06	Ni_4–O_{20}: 2.22	Ni_2–O_{10}: 2.06	Ni_4–O_{20}: 2.08

Note: The atomic index is the same as that in Fig. S9.

Table S5 Comparison of the cell voltage, current density and stability test time of the cell in the previous works and this work

No.	Cell	Cell Voltage (V)	Current density (mA cm^{-2})	Electrolytes	Raw materials	Stability test time (h)	Refs.
1	$Co(OH)_2$@HOS || $Co(OH)_2$@HOS	1.56	10	1 M KOH	methanol	20	Adv. Funct. Mater. **2020**, *30*, 1909610
2	Co_xP@NiCo–LDH || Co_xP@NiCo–LDH	1.6	10	1 M KOH	methanol	20	J. Energy Chem. **2020**, *50*, 314
3	$Ni(OH)_2$/NF || $Ni(OH)_2$/NF	1.56	20	1 M KOH	methanol	20	Appl. Catal. B **2021**, *281*, 119510
4	MnO_2/NF || MnO_2/NF	1.40	10	1 M KOH	urea	20	Angew. Chem. Int. Ed. **2016**, *55*, 3804
5	NiMoO–H_2 || NiMoO–Ar	1.55	100	1 M KOH	urea	50	Energy Environ. Sci. **2018**, *11*, 1890
6	Ni–WOx || Ag NPs	2.16	100	1 M KOH	urea	10	Angew. Chem. Int. Ed. **2021**, *60*, 10577
7	Ni_3N–350/NF|| Ni_3N–350/NF	1.51	100	1 M KOH	urea	25	ACS Sustain. Chem. Eng. **2020**, *8*, 7414–7422
8	O–NiMoP/NF || O–NiMoP/NF	1.40	20	1 M KOH	urea	10	Adv. Funct. Mater. **2021**, *31*, 2104951
9	Ni–NiO–$Mo_{0.84}Ni_{0.16}$/NF|| Ni–NiO–$Mo_{0.84}Ni_{0.16}$/NF	1.77	150	1 M KOH	urea	60	ACS Sustain. Chem. Eng. **2020**, *8*, 7174–7181
10	W–NiS_2/MoO_2@CC|| W–NiS_2/MoO_2@CC	1.4	100	1 M KOH	urea	24	Chem. Eng. J. **2022**, *432*, 134274
11	1%Cu:α–$Ni(OH)_2$|| 1%Cu:α–$Ni(OH)_2$	1.6	11	1 M KOH	urea	40	J. Mater. Chem. A **2019**, *7*, 13577-13584
12	NiClO–D|| Pt/C	1.7	100	1 M KOH	urea	1	Angew. Chem. Int. Ed. **2019**, *58*, 16820
13	CoMn/$CoMn_2O_4$|| CoMn/$CoMn_2O_4$	1.68	100	1 M KOH	urea	17	Adv. Funct. Mater. **2020**, *30*, 2000556
14	Ni(OH)2/CuO NWs/CF || Pt/C/NF	1.42	20	1 M KOH	urea	150	Small method **2022**, *6*, 2101017
15	Ni_4Mo–MoO_2 || LC–$Ni(OH)_2{\cdot}xH_2O$	2	>500	6 M KOH	methanol	50	This work

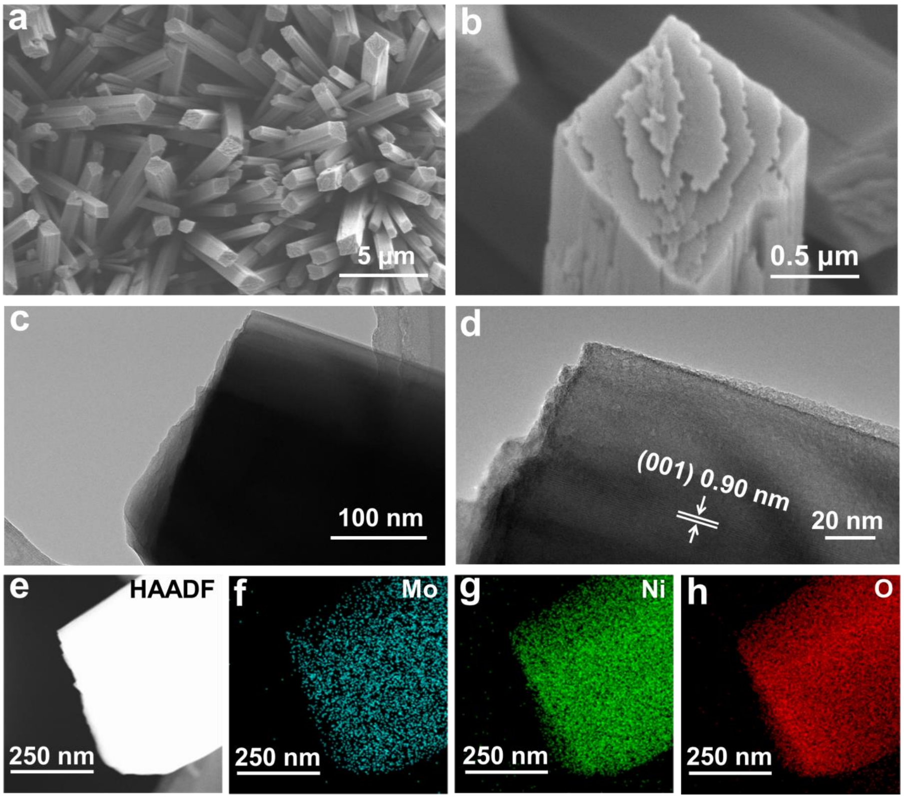

Fig. S1 a, b SEM image of the $NiMoO_4 \cdot$ 0.75 H_2O. **c, d** TEM image of the $NiMoO_4 \cdot$ 0.75 H_2O. **e** HADDF image of the $NiMoO_4 \cdot$ 0.75 H_2O. **f, g** Elemental mapping of the $NiMoO_4 \cdot$ 0.75 H_2O

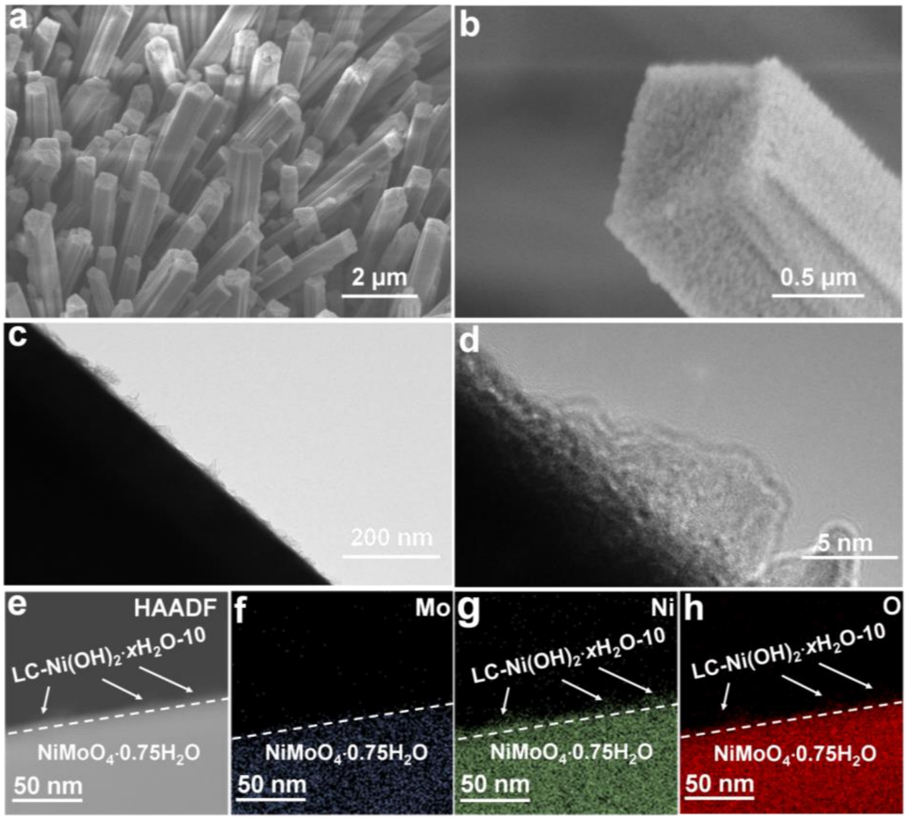

Fig S2. a, b SEM image of LC–$Ni(OH)_2 \cdot xH_2O$–10. **c, d** TEM image of LC–$Ni(OH)_2 \cdot xH_2O$–10. **e** HADDF image of LC–$Ni(OH)_2 \cdot xH_2O$–10. **f, g** Elemental mapping of LC–$Ni(OH)_2 \cdot xH_2O$–10

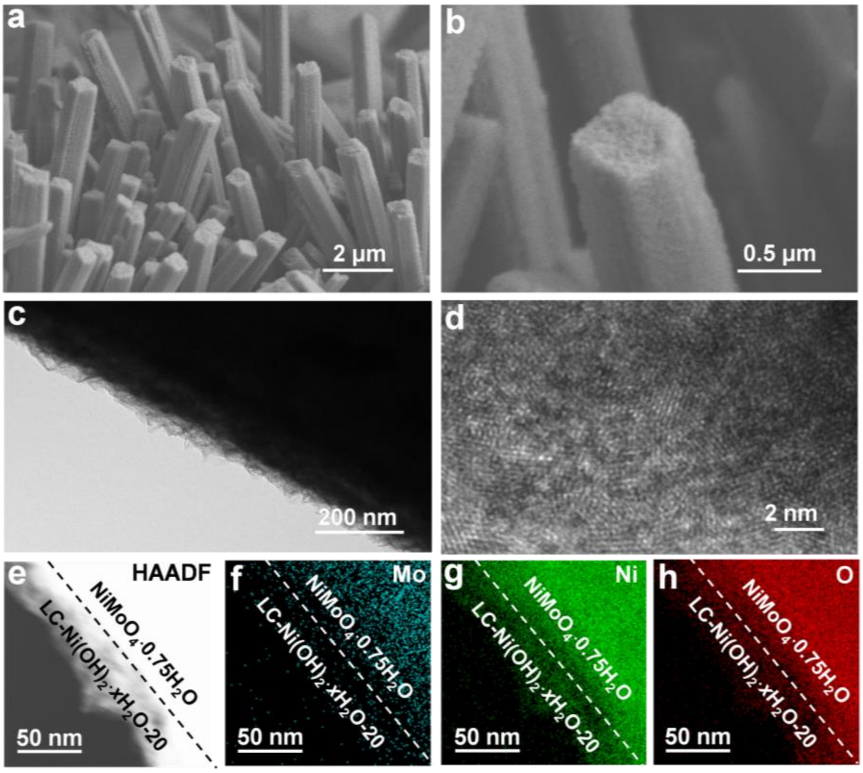

Fig. S3 a, b SEM image of LC–Ni(OH)$_2$·xH$_2$O–20. **c, d** TEM image of LC–Ni(OH)$_2$·xH$_2$O–20. **e** HADDF image of LC–Ni(OH)$_2$·xH$_2$O–20. **f, g** Elemental mapping of LC–Ni(OH)$_2$·xH$_2$O–20

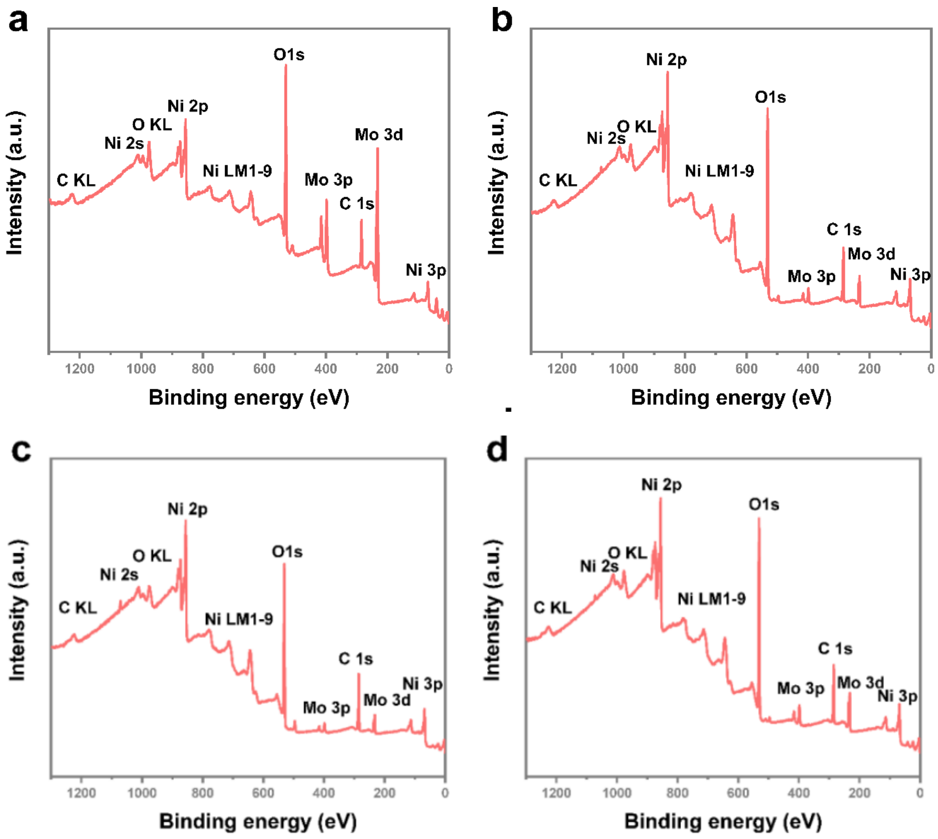

Fig. S4 XPS survey spectra of **a** NiMoO$_4$· H$_2$O, **b** LC–Ni(OH)$_2$·xH$_2$O–10, **c** LC–Ni(OH)$_2$·xH$_2$O–20, **d** LC–Ni(OH)$_2$·xH$_2$O

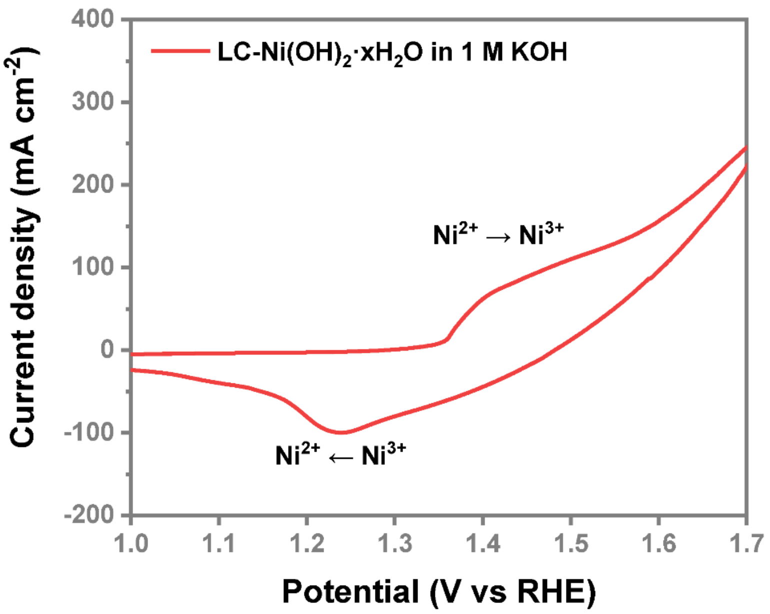

Fig. S5 Cycle voltametric curve of LC-Ni(OH)$_2$·xH$_2$O in 1M KOH

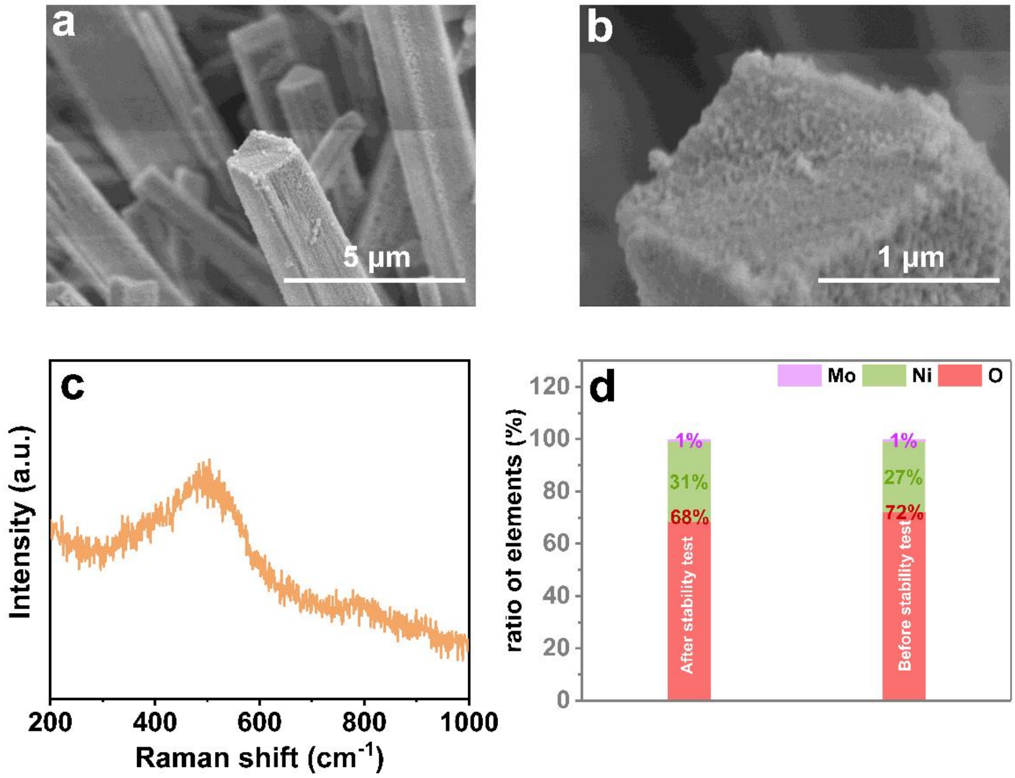

Fig. S6 a, b SEM image and **c** Raman spectra of LC–Ni(OH)$_2$·xH$_2$O after 100000s stability test. **d** ICP-OES for the ratio of Mo, Ni and O of LC–Ni(OH)$_2$·xH$_2$O before and after 100000s stability

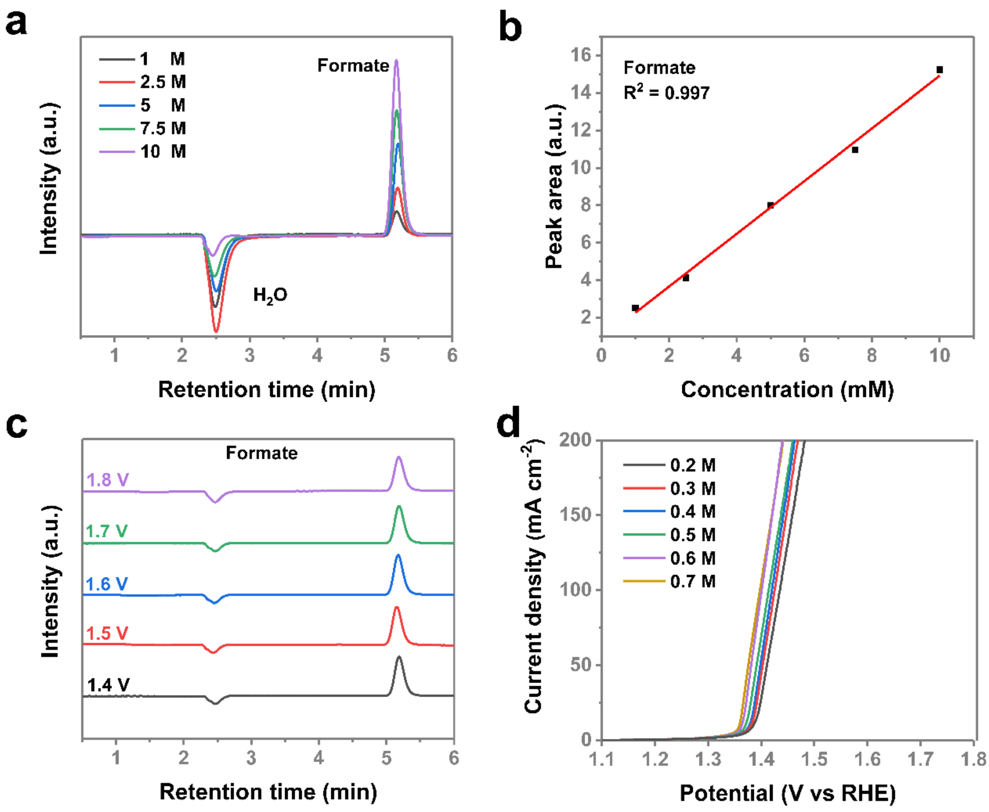

Fig. S7 a The standard curve for formate at the concentration of 1.0, 2.5, 5.0, 7.5 and 10.0 M. **b** The correlation of the concentration between concentration(mM) and the peak area (a.u.) for formate. **c** The ion chromatography spectra for formate at different potentials. **d** LSV curve for LC–$Ni(OH)_2{\cdot}xH_2O$ in 1 M KOH with different methanol concentrations (0.2-0.7M)

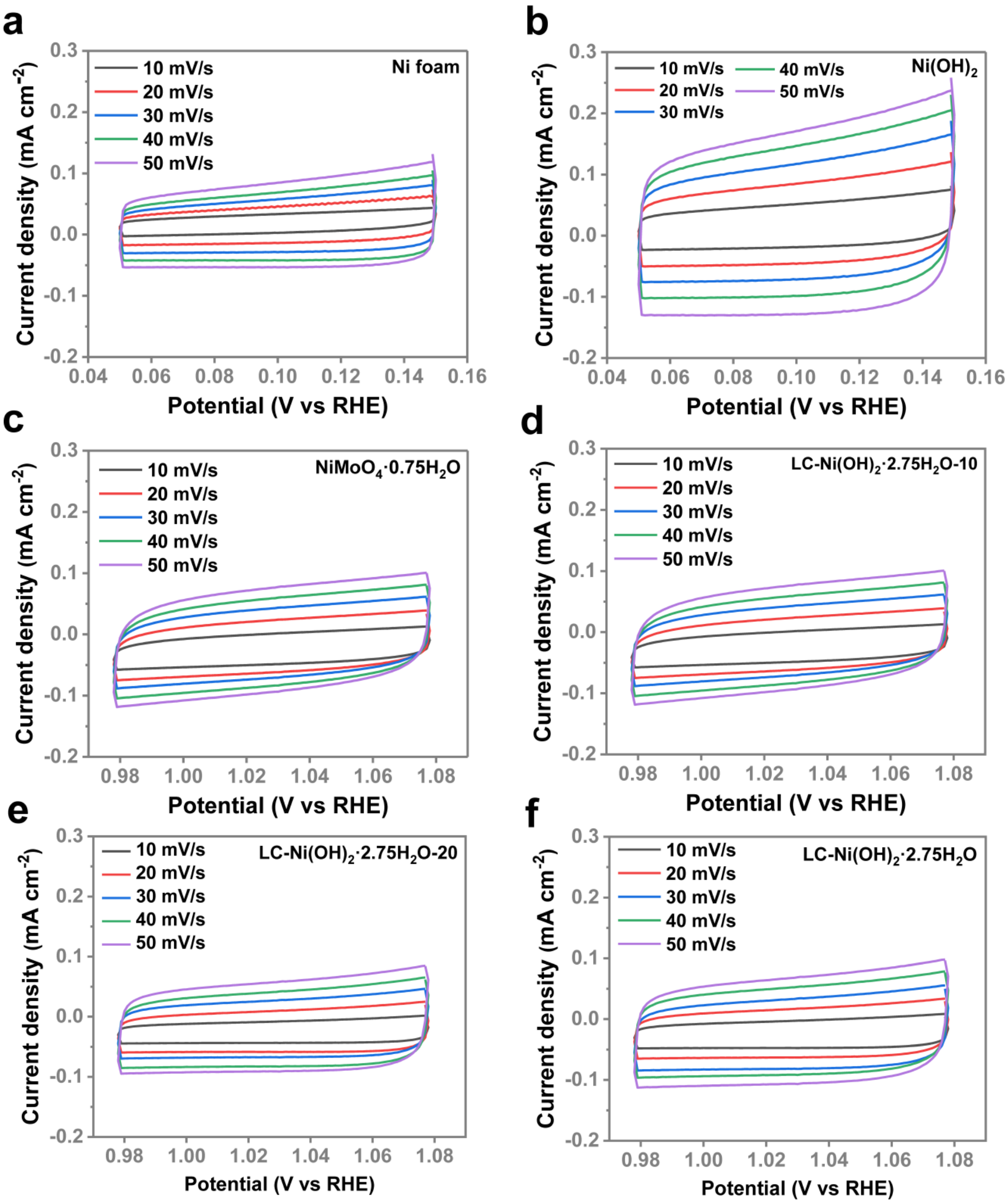

Fig. S8 CV curve of **a** Ni foam, **b** $Ni(OH)_2$, **c** $NiMoO_4 \cdot$ $0.75H_2O$, **d** LC–$Ni(OH)_2 \cdot xH_2O$–10, **e** LC–$Ni(OH)_2 \cdot xH_2O$–20, **f** LC–$Ni(OH)_2 \cdot xH_2O$ at different scan rate

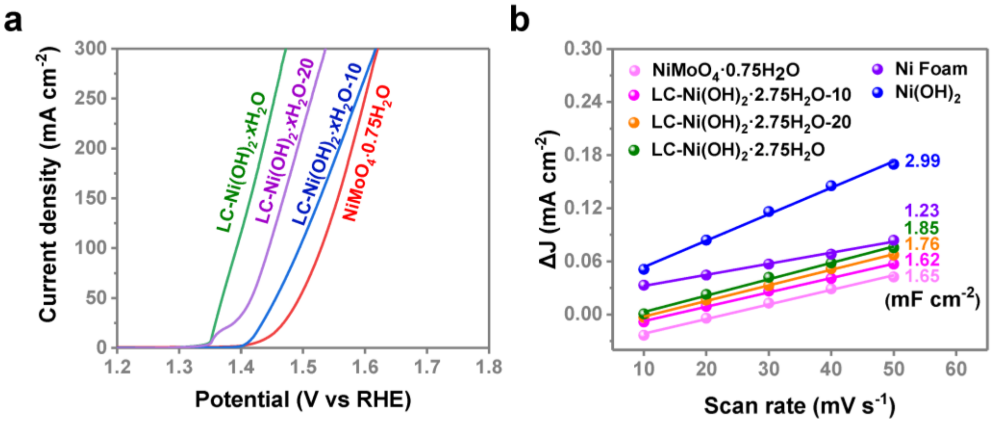

Fig. S9 a LSV curve of different reconstruction time of LC–$Ni(OH)_2 \cdot xH_2O$ catalysts in 1 M KOH and 0.5 M methanol. **b** Linear fitting of the electrochemically active surface area for different samples

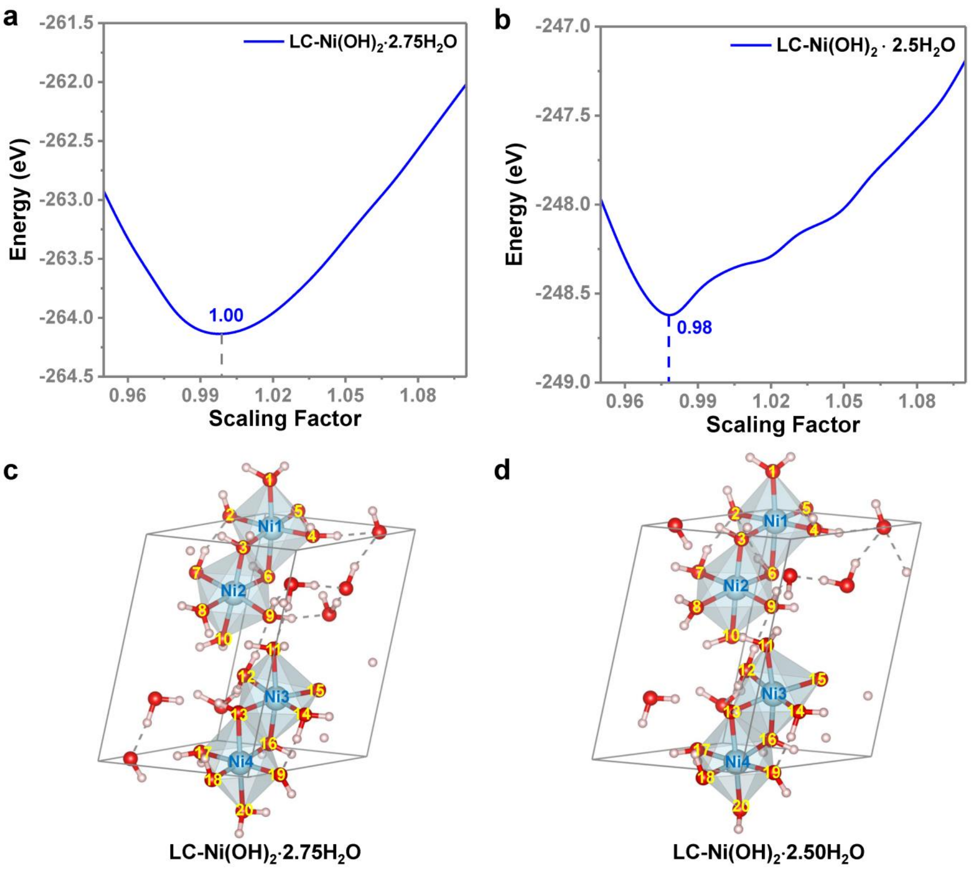

Fig. S10 Optimization of lattice parameters for **a** LC–Ni(OH)$_2$·2.75H$_2$O and **b** LC–Ni(OH)$_2$·2.50H$_2$O. Optimized structures for **c** LC–Ni(OH)$_2$·2.75H$_2$O and **d** LC–Ni(OH)$_2$·2.50H$_2$O

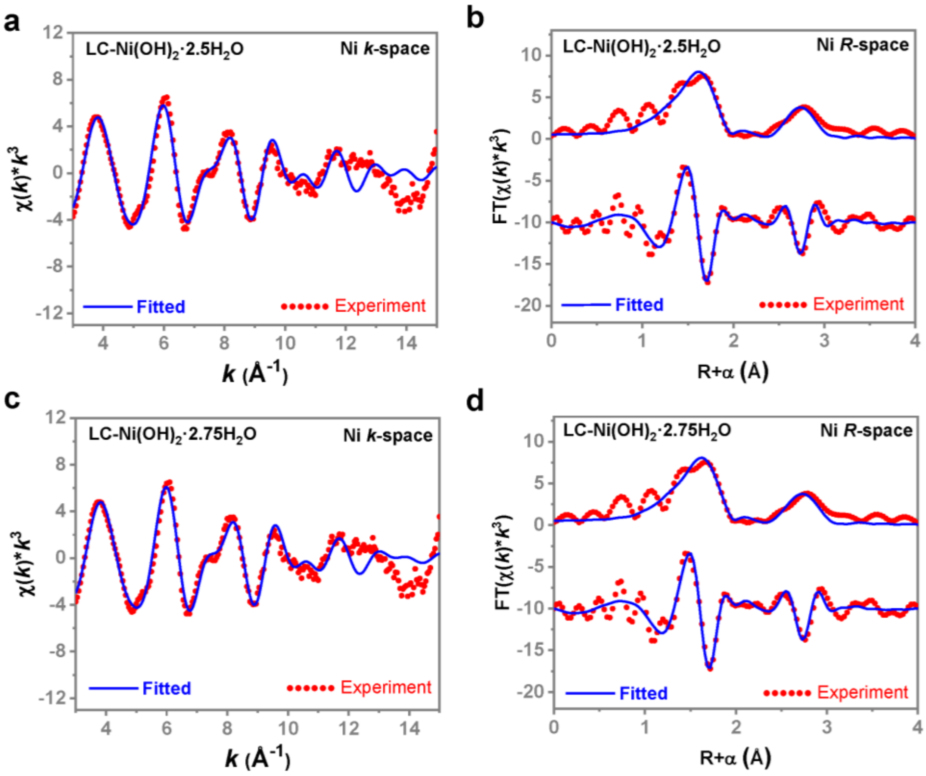

Fig. S11 EXAFS fitting results (line) and experiment data (point) of Ni K edge in *k*-space and *R*-space for different samples

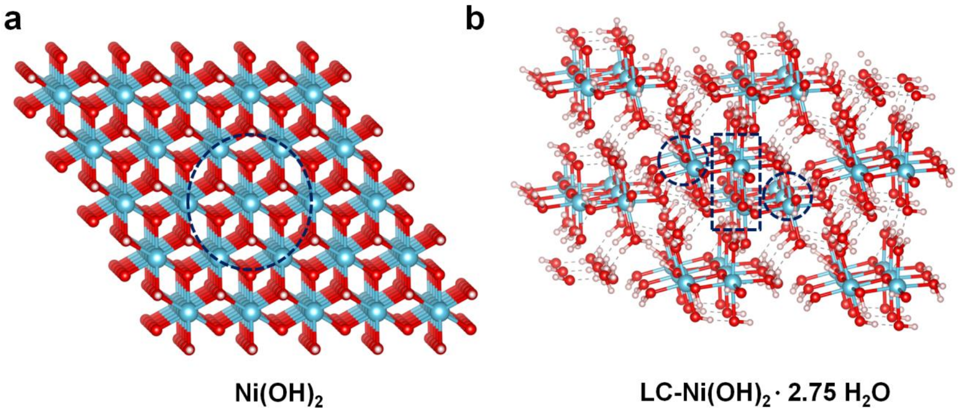

Fig. S12 a Illustration of $Ni(OH)_2$ with 6 fold Ni‑Ni coordination as indicated by the cycle. **b** Illustration of LC–$Ni(OH)_2 \cdot 2.75H_2O$ with average 1.5 fold Ni‑Ni coordination. The cycle indicates 2 fold coordination whereas the cycle indicates 1 fold Ni‑Ni coordination

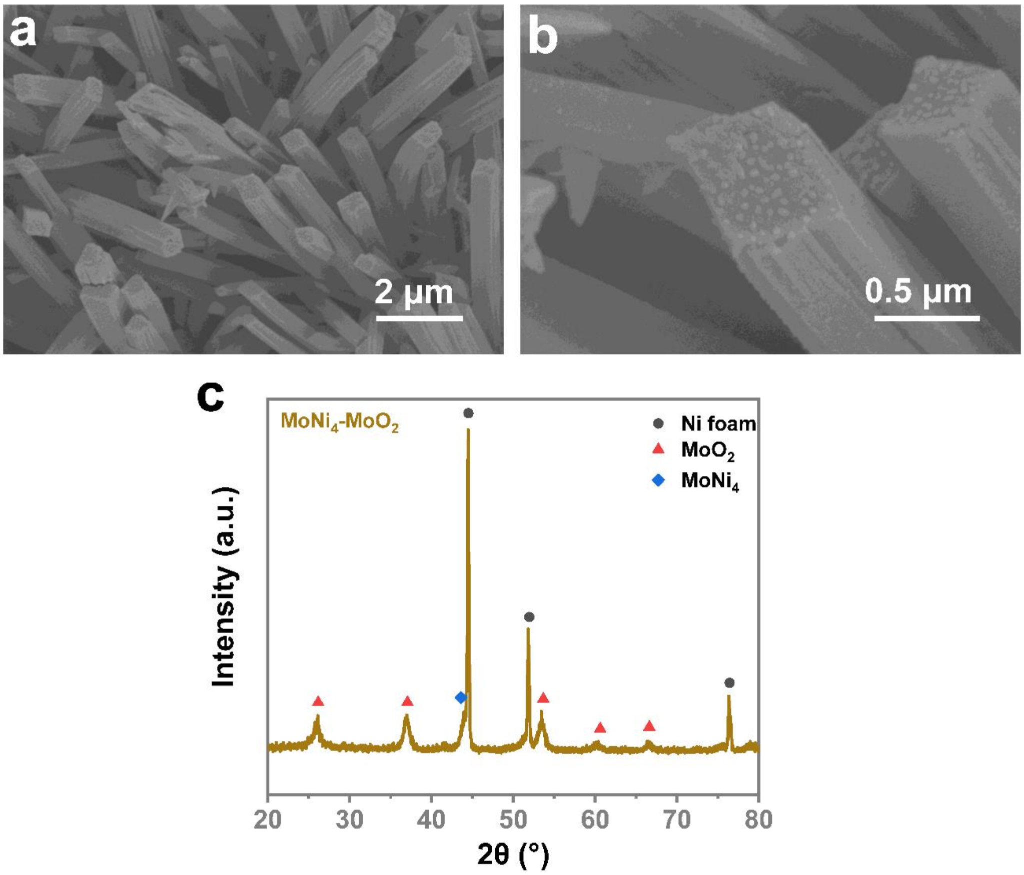

Fig. S13 a, b SEM image of Ni_4Mo–MoO_2. **c** XRD of Ni_4Mo–MoO_2

Supplementary References

[S1]B. Ravel, M. Newville, ATHENA, ARTEMIS, HEPHAESTUS: data analysis for X-ray absorption spectroscopy using IFEFFIT. J. Synchrotron Rad. **12**, 537-541 (2005). https://doi.org/10.1107/S0909049505012719
